# Proteomics approach to investigating osmotic stress effects on pistachio

**DOI:** 10.3389/fpls.2022.1041649

**Published:** 2023-01-25

**Authors:** Rambod Pakzad, Foad Fatehi, Mansour Kalantar, Mahmood Maleki

**Affiliations:** ^1^ Department of Plant Breeding, Yazd Branch, Islamic Azad University, Yazd, Iran; ^2^ Department of Agriculture, Payame Noor University (PNU), Tehran, Iran; ^3^ Department of Biotechnology, Institute of Science and High Technology and Environmental Sciences, Graduate University of Advanced Technology, Kerman, Iran

**Keywords:** dehydration, osmotic stress, pistachio, proteomics, stress response

## Abstract

Osmotic stress can occur due to some stresses such as salinity and drought, threatening plant survival. To investigate the mechanism governing the pistachio response to this stress, the biochemical alterations and protein profile of PEG-treated plants was monitored. Also, we selected two differentially abundant proteins to validate *via* Real-Time PCR. Biochemical results displayed that in treated plants, proline and phenolic content was elevated, photosynthetic pigments except carotenoid decreased and MDA concentration were not altered. Our findings identified a number of proteins using 2DE-MS, involved in mitigating osmotic stress in pistachio. A total of 180 protein spots were identified, of which 25 spots were altered in response to osmotic stress. Four spots that had photosynthetic activities were down-regulated, and the remaining spots were up-regulated. The biological functional analysis of protein spots exhibited that most of them are associated with the photosynthesis and metabolism (36%) followed by stress response (24%). Results of Real-Time PCR indicated that two of the representative genes illustrated a positive correlation among transcript level and protein expression and had a similar trend in regulation of gene and protein. Osmotic stress set changes in the proteins associated with photosynthesis and stress tolerance, proteins associated with the cell wall, changes in the expression of proteins involved in DNA and RNA processing occur. Findings of this research will introduce possible proteins and pathways that contribute to osmotic stress and can be considered for improving osmotic tolerance in pistachio.

## Introduction

Since plants are sessile, they could not change their location and are continuously subjected to various stresses that threaten their survival. Osmotic stress, which results from abiotic stresses such as salinity, drought, and cold, and is one of the most common stresses in nature, is caused by a decrease in water potential in the environment around the roots (Xiong and Zhu, 2002; Zang and Komatsu, 2007; Toorchi et al., 2009), which limits the plant’s ability to absorb water and restricts water accessibility (Zang and Komatsu, 2007). Osmotic stress appears in various morphological, physiological, and biochemical dimensions in the plant. Tolerance to stress is a complicated phenomenon. To deal with this stress, plants trigger a variety of response mechanisms that require three steps of stress recognition, signal transduction, and the generation of related response components (Zang and Komatsu, 2007; Zhou et al., 2012). These responses enable plants to save water and reprogram cell metabolism for adaptation to stress (Ngara et al., 2018).

Plant survival against stress requires the rearrangement of many molecular processes and reregulation of many genes. Examination of mRNA expression is not sufficient to predict the events that occur in the plant during exposure to stress because there is a low correlation between the abundance of mRNAs and proteins (Wang et al., 2020). Moreover, proteins play important roles in all cellular processes such as gene regulation, transcription, translation, cell detoxification, protection of macromolecules, and osmotic adjustment (Pasaribu et al., 2021). Hence, studying the expression of proteins provides us with more information about plant behavior under stress. Numerous studies show that proteomics is a beneficial tool for analyzing osmotic stress induced changes. Zang and Komatsu (2007) showed that the accumulation of 15 proteins under stress was altered in rice, most of which were involved in lipid accumulation, proteasome regulatory pathway, and glyoxalase system. Applying osmotic stress, in addition to altering the expression of 37 proteins, including affeoyl-CoA-O-methyltransferase and 20S proteasome alpha subunit A, led to reduced root and hypocotyl lengths in soybean (Toorchi et al., 2009). It has been reported that some main stress-responsive genes and proteins involved in ROS scavenging, phytohormone and protein metabolism, membrane stability, transport and signaling were active under osmotic stress (Zhang and Shi, 2018; Wang et al., 2020).

Iran, as an origin area of pistachio and its largest producer, is located in the arid regions, where environmental stresses which cause osmotic stress, constantly threaten agriculture (Esmaeilpour et al., 2015). According to the report of the Food and Agriculture Organization (FAO), countries such as Iran, America, Turkey, China and Syria respectively have the largest production of pistachios in the world (FAO, 2020). Pistachio is one of the most important strategic products of Iran, which has decreased in recent years due to the increase of osmotic stresses such as salinity stresses (Rahimi et al., 2021). According to the FAO report, the amount of production of this valuable product in Iran has decreased from 575 thousand tons in 2016 to 190 thousand tons in 2020 (FAO, 2020). Iran is the principal exporter of pistachio crop in the world, which recently its production due to over salinity water and soil has been reduced. According to this fact, in the present study, our aim is to identify important pathways related to osmotic stress through investigating changes of the proteome profiling of pistachio leaves using 2DE-MS under osmotic stress.

## Materials and methods

The seeds of *Pistacia vera* L. cv. Akbari were obtained from the Iranian Pistachio Research Institute (IPRI), Rafsanjan, Iran. The seeds were soaked in water for 24h and germinated for a week in 9cm petri dishes with double layers of Whatman filter paper. The germinated seeds were sown in 5L pots containing perlite and irrigated by Hoagland solution for 10 weeks in a controlled greenhouse (25°C, 16h light/8h dark photoperiod with 30% relative humidity) (Esmaeilpour et al., 2015). Then, plants were divided into two groups, control group and osmotic treatment group, each treatment with three replicates (three plants per pots and two pots per replication). Pre-experiments was conducted to select the osmotic treatment. The osmotic treatment (-1.5 MPa) was applied by adding polyethylene glycol 6000 (PEG6000) to Hoagland solution as described by Khoyerdi et al. (Khoyerdi et al., 2016) and maintained for two weeks. The fully expanded leaves from the tip of each plant were frozen in liquid nitrogen prior to being stored at -70°C for physiological measurements and proteomics study.

### Biochemical assays

For three biological replicates of each treatment, proline was quantified following Carillo and Gibon (2011). Determining the concentration of phenolic compounds was performed according to Ainsworth and Gillespie (2007). Malondialdehyde (MDA) content were measured based on the study of Velikova et al. (Velikova et al., 2000). Photosynthetic pigment’s assay (chlorophyll a, chlorophyll b, and carotenoids) was carried out according to the method of Lichtenthaler and Buschmann (2001).

### Protein extraction

According to modified Hurkman and Tanaka (1986) method, after homogenizing 500mg of fresh leaves in liquid nitrogen for three biological replicates of each treatment, 1 mL of cold extraction buffer comprising of 20mM Tris−HCl, (pH 7.5), 1mM EGTA, 1mM PMSF, and 1mM DTT was prepared and added. Then, the sample was incubated at 4°C for 90 min and centrifuged at 20,000×g for 45 min. Four volumes of cold acetone containing 0.08% β-mercaptoethanol and 12% TCA was added to the supernatant as it was incubated at -10°C for 15h. After that, the sample was centrifuged at 20,000×g for 45min. The pellet was washed by cold acetone including 0.08% β-mercaptoethanol seven times at -10°C for 4h and then lyophilized. Finally, the pellet was resolved in lysis buffer comprising of 7M urea, 2M thiourea, 4% CHAPS, 35mM TRIS−HCl, 1% w/v DTT, and 1% v/v Ampholyte, pH 3.5–10) and incubated at 25°C for 1h and then centrifuged at 12,000×g for 15min. The supernatant containing proteins was stored at -80°C. Proteins amounts were assayed by Bradford (1976) method.

### Detection of proteins by 2-dimensional gel electrophoresis

120µg protein was added to 320µg rehydration buffer including 8M urea, 2% CHAPS, 0.018M DTT, 2% IPG buffer (pH 3–10), and 0.002% bromophenol blue. Rehydration buffer was loaded to 17 cm IPG linear gradient strips (Bio-Rad) with pH 4–7 in a rehydration tray at 25°C for 12–16h. Isoelectric focusing (IEF) was carried out on a Multiphor II electrophoresis system (Amersham Pharmacia Biotech) at 20°C pursuant to the following conditions: 150Vh at 0–300 V, 300Vh at 300–500 V, followed by 2000Vh at 500-3500 V and finally 39,500Vh at 3500V. A maximum of 50µA per strip was used for the electric current. Equilibrium buffer comprising of 50mM Tris-HCl (pH 8.8), 6M urea, 30% glycerol (v/v), 2% SDS, 1% DTT, and bromophenol blue was used for balancing IEF strips for 15 min. Afterwards, strips were put onto 12.5% SDS-PAGE gels on Protein II Xi Cell (Bio- Rad) apparatus. Gels were stained using silver nitrate according to Blum et al. (Blum et al., 1987) protocol. After staining, the gels were scanned using Bio-Rad’s GS800 densitometer and then converted into TIF format using PDQuest software. Melanie software (version 7) was used for quantitative and qualitative evaluation of protein spots in different treatments (Fatehi et al., 2012; Fatehi et al., 2013). Only spots with reproducible alternations (at least 1.5-fold change) in three biological replicates were used in further analyzes.

### In gel digestion and of protein Identification by MALDI/TOF/TOF MS

In-gel digestion and mass spectrometry were carried out according to Pakzad et al. (Pakzad et al., 2019). Briefly, هspot were manually excised from the gels and destined for 1 h at 28 C by fresh wash solution (50% acetonitrile 50 mM ammonium bicarbonate (50:50 v/v)). Then washing solution were eliminated and spots dried for 30 min at 37°C. Protein reclamation and alkylation were carried out by 10 mM dithiotreitol (DTT) and 55 mM iodoacetamide (IAA), respectively, and then tryptic digestion were done in 50mM ammonium bicarbonate (pH 8) using MassPREP automated digester station (PerkinElmer). Peptides were extracted using a solution containing 2% acetonitrile and 1% formic acid and lyophilized. Using a solution including of 0.1% TFA (trifluoroacetic acid) and 10% acetonitrile, lyophilized peptides were solved. The peptide mixed in 5 mg/mL of α-cyano-4-hydroxycinnamic acid (CHCA) (MALDI matrix), 50% acetonitrile, 6mM ammonium phosphate monobasic and 0.1% trifluoroacetic acid. The Mass Spectrometry information were obtained using an AB Sciex 5800 TOF/TOF System, MALDI/TOF/TOF (Framingham, MA, USA) with a 349 nm Nd : YLF OptiBeam On-Axis laser.

### Protein characterization and classification

Mass spectrometry data was analyzed by MASCOT software (Version 2.2, Matrix Science, London, UK) against Swiss-Prot. Protein properties were gained from UniProt database (https://www.uniprot.org/). More information about the function of proteins was obtained from the scientific literature.

### Bioinformatics analysis

The Gene Ontology (GO) analysis of identified proteins were investigated *via* Uniprot (http://www.uniprot.org) and string database (https://string-db.org). Protein–protein interaction was evaluated by the search tool for interactions of chemicals (STITCH) (http://stitch.embl.de).

### Validate identified proteins using quantitative real-time PCR

Based on the proteomics results, we validated three identified proteins *via* q-PCR. Total RNA was extracted from control and treated pistachio leaf by RNX – plus kit (Sinaclon, Iran). Synthesis of cDNA and qRT-PCR were done as illustrated by Sadeghi, Mirzaei (Sadeghi et al., 2022). Primers were designed using Primer 3 software (**Table 1**). Three biological and technical replicates were considered for each sample. The *EF1α* gene was considered as an endogenous control (Moazzam Jazi et al., 2016) and normalization of the CT value of each gene was done by REST software (Relative Expression Software Tool). Change of transcription levels were quantified through the Pfaffl method (Pfaffl et al., 2002).

**Table 1:** The sequence of primers designed in this study.

Gene	Forward and Reverse primers sequence (5′ - 3′)	GC%	Annealing temperature (°C)	Amplicon size (bp)
Actin	F: GTCAGCCACACTGTCCCCAT	60	62.13	91
	R: GGGCGTCAGTAAGGTCACGA	60	61.87	
Catalase isozyme 1	F: CAGGCGGACAAATCACTGGG	60 55	61.31 61.19	135
	R: ACAGCAGTCATCCTTCCCGT			
Abscisic acid receptor PYL9	F: CCAAACCCAACCCAAAGGTGA	52.38	60.97	131
	R: CTCTGGGCTCGTGTCTGTGA	60	61.53	
Aspartokinase 2	F: AGTGAGTTGTGAGGGAGCGA	55	60.83	132
	R: CTCTCAGCAGAGGACACGGA	60	60.96	

### Statistical analysis

To evaluate the significant differences between mean values of control and osmotic treatment t-test were performed using SAS software v9.1 (SAS Institute Inc., Cary, NC, USA). The measurements are presented as mean ± standard deviation (SD) of 18 samples.

## Results

### Biochemical parameters

**Figure 1:**
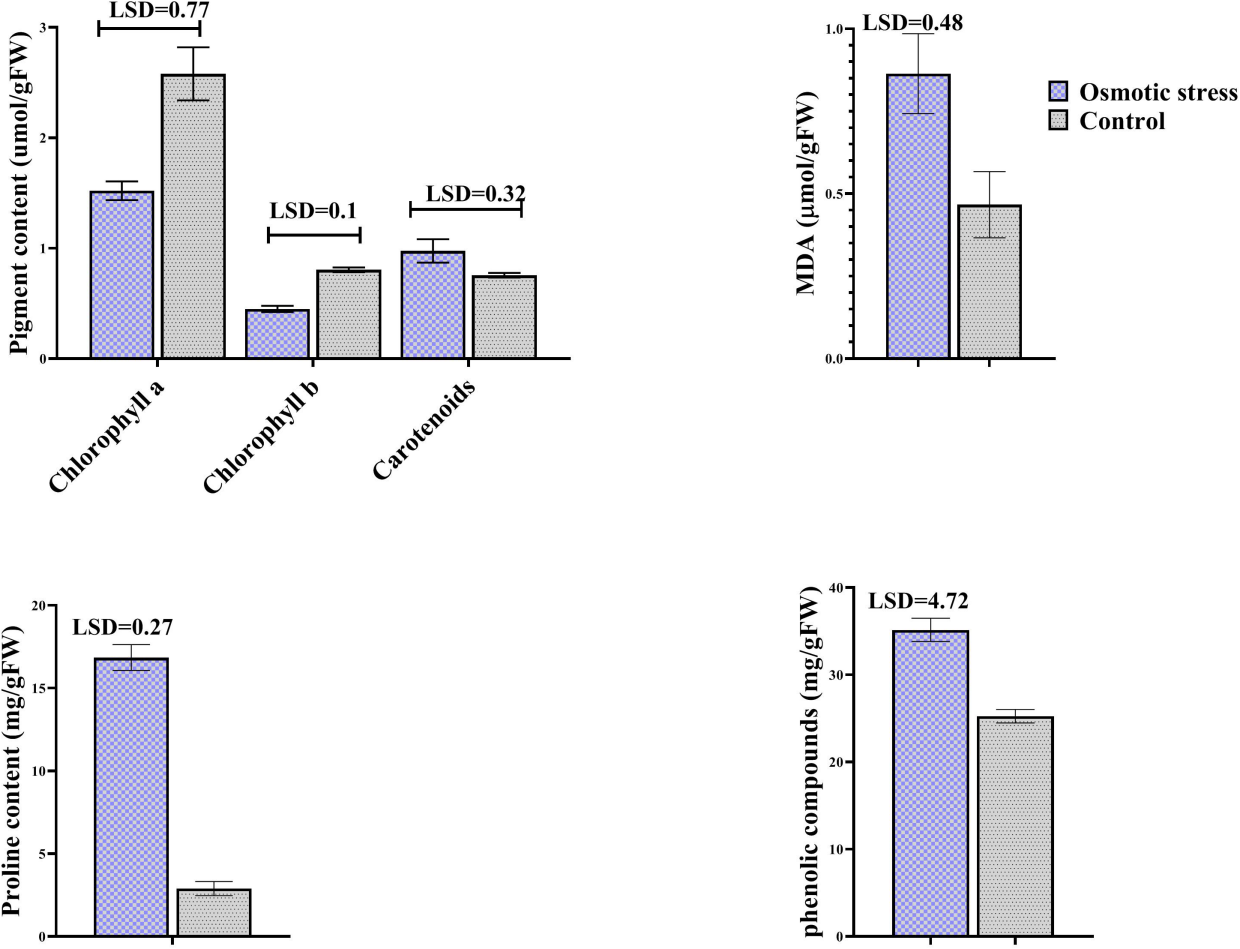
The effect of osmotic stress on photosynthetic pigments, MDA, proline and phenolic compounds in leaves of pistachio under osmotic stress compared with control. Bars indicated the SD (n = 3). Statistic were carried out at p = 0.05 according to the t test.

MDA concentration was assayed as a lipid peroxidation product. Osmotic stress did not change its concentration, while it increased the level of phenolic compounds. Proline content was dramatically increased when osmotic stress was applied. Stress affected photosynthetic pigments except carotenoid, so that chlorophylls were degraded under stress (**Figure 1**).

### Identification of differentially regulated proteins

**Figure 2:**
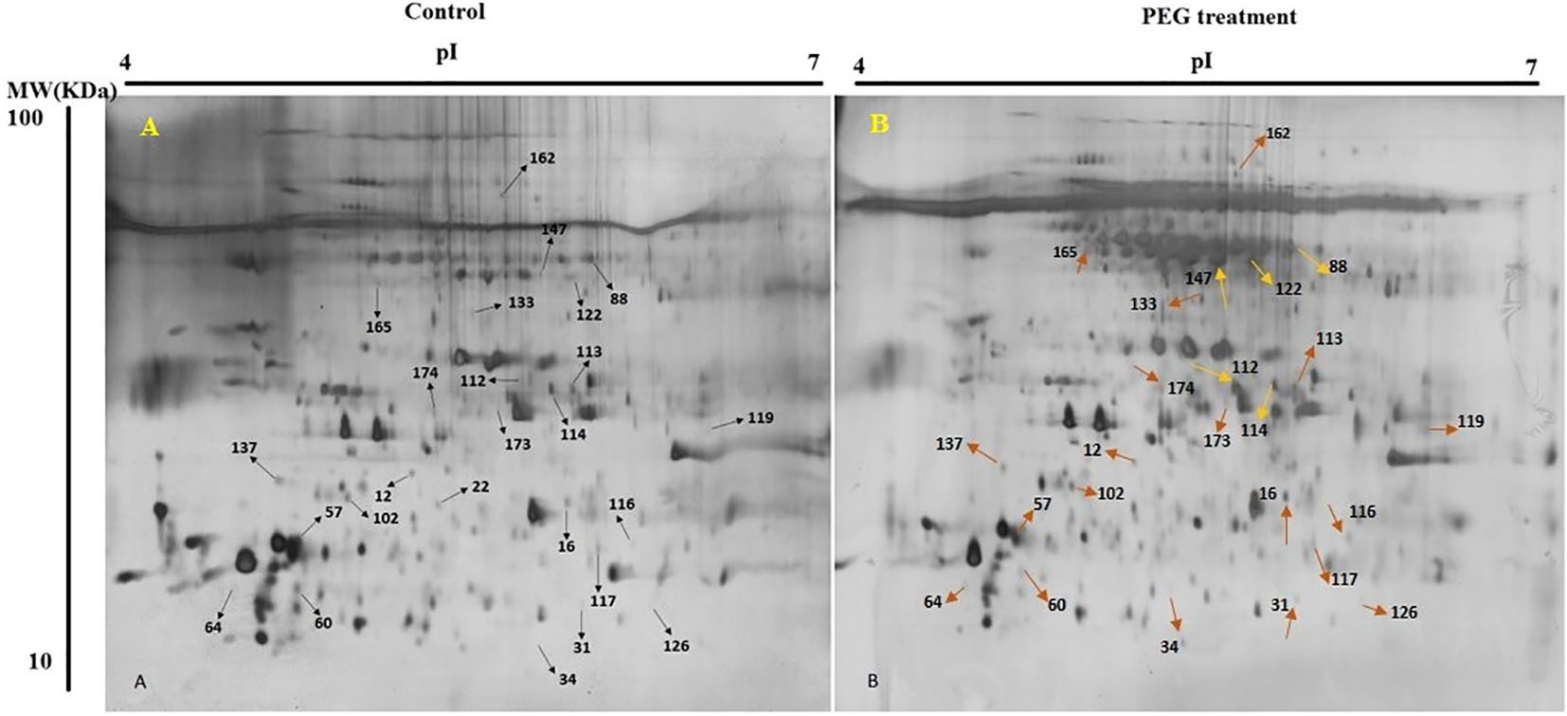
2-DE gel images of protein profiles from pistachio leaves. Control **(A)** and PEG treatment **(B)**. 17cm IPG strip (pH 7-14) was used for loading proteins and SDS-PAGE was done with a 12% gel. Gel was stained using CBB G-250. Proteins with differential regulation levels are marked by arrows.

In present study, for finding the impact of osmotic stress, pistachio seedlings were treated with PEG6000 to apply osmotic stress for two weeks. Then, proteins of three biological replicates for treatment and control were extracted from leaves and separated by 2-DE (**Figure 2**). Silver nitrate and Melanie software were used for gel staining and analyzing, respectively. Only differentially accumulated protein spots that represented reproducible alterations were used for further analysis by MS.

Out of 280 detected spots, 25 protein spots were significantly altered in response to osmotic stress, accounting for about 8.9% of the detected spots. Among them, only four proteins (spots 57, 64, 102, and 137) were down-regulated and the others were up-regulated (**Table 2**).

**Table 2:** Protein properties of differentially expressed proteins of pistachio leaves under osmotic stress.

Annotations	Spot No.	Accession No.	Score	Coverage%	MW (Da)	pI	Accumulation Status
Photosynthesis and metabolism							
Ribulose bisphosphate carboxylase large chain	57	P28458	58	12	52115	5.91	_*
Ribulose bisphosphate carboxylase small subunit	64	P07180	58	28	20446	6.73	_
Oxygen-evolving enhancer protein 1	102	P85194	57	29	34487	5.4	_
Photosystem I assembly protein Ycf4	137	Q8WI09	60	24	21398	9.83	_
Aspartokinase 2, chloroplastic	133	O23653	62	22	60137	6.31	+*
Glyceraldehyde-3-phosphate dehydrogenase A	113	P19866	52	28	43338	7.62	+
Putative cytochrome c oxidase subunit II PS17	31	P84733	41	100	1707	9.62	+
Thiosulfate/3-mercaptopyruvate sulfurtransferase 1	112	O64530	48	16	42152	5.95	+
Cytosolic sulfotransferase 4	12	Q8RUC1	54	35	32368	8.68	+
Stress response							
Abscisic acid receptor PYL9	116	Q84MC7	41	31	21173	5.89	+
Phospholipase D alpha 3	122	P58766	41	7	93931	6.36	+
18.1 kDa class I heat shock	117	P19037	40	25	18123	6.77	+
Catalase-2	119	P25819	63	24	57237	6.63	+
Catalase isozyme 2	173	Q9XHH3	43	19	57141	7.71	+
Probable serine/threonine-protein kinase CST	34	P27450	51	19	46482	9.58	+
DNA and RNA processing							
DEAD-box ATP-dependent RNA helicase 7	16	Q39189	62	24	73187	9.29	+
DNA repair protein RAD50	162	Q9SL02	68	22	153632	5.98	+
Replication protein A 70 kDa DNA-binding subunit D	174	Q9FME0	58	33	70676	6.1	+
Protein argonaute MEL1	60	Q851R2	66	8	117987	9.34	+
Cell wall biosynthesis							
Prolyl 4-hydroxylase 5	147	Q24JN5	42	11	32842	7.75	+
Probable galacturonosyltransferase 3	165	Q0WQD2	58	15	78178	7.27	+
Transporting and movement							
Putative aluminum-activated malate transporter 11	22	Q3E9Z9	38	24	17149	9.55	+
Kinesin-like protein KIN-7H	88	F4JZ68	57	27	122375	5.53	+
Signal transduction							
Guanine nucleotide-binding protein alpha-1 subunit	126	P18064	59	32	44860	5.96	+
Other							
F-box/kelch-repeat protein At3g17530	114	Q9LUP5	48	26	44807	7.43	+

*+: Up-regulated expression, _: Down-regulated expression, ND, no data.

MALDI-TOF/TOF MS was applied to distinguish possible identities of differentially expressed spots. Mascot search engine searched the Swiss-Prot database, while a higher score as well as higher sequence coverage was our criteria for selection.

The calculated pIs of nearly half of the identified proteins were in the acidic pH range and those of the other half were in the neutral and alkaline pH range. 64% of them were distributed in the range of 10,000 – 100,000 Da. whereas, monoisotopic mass of protein 31 was below 10,000 Da and those of proteins 60, 88, and 162 were above 100,000 Da.

### Functional classification of differentially regulated proteins

**Figure 3:**
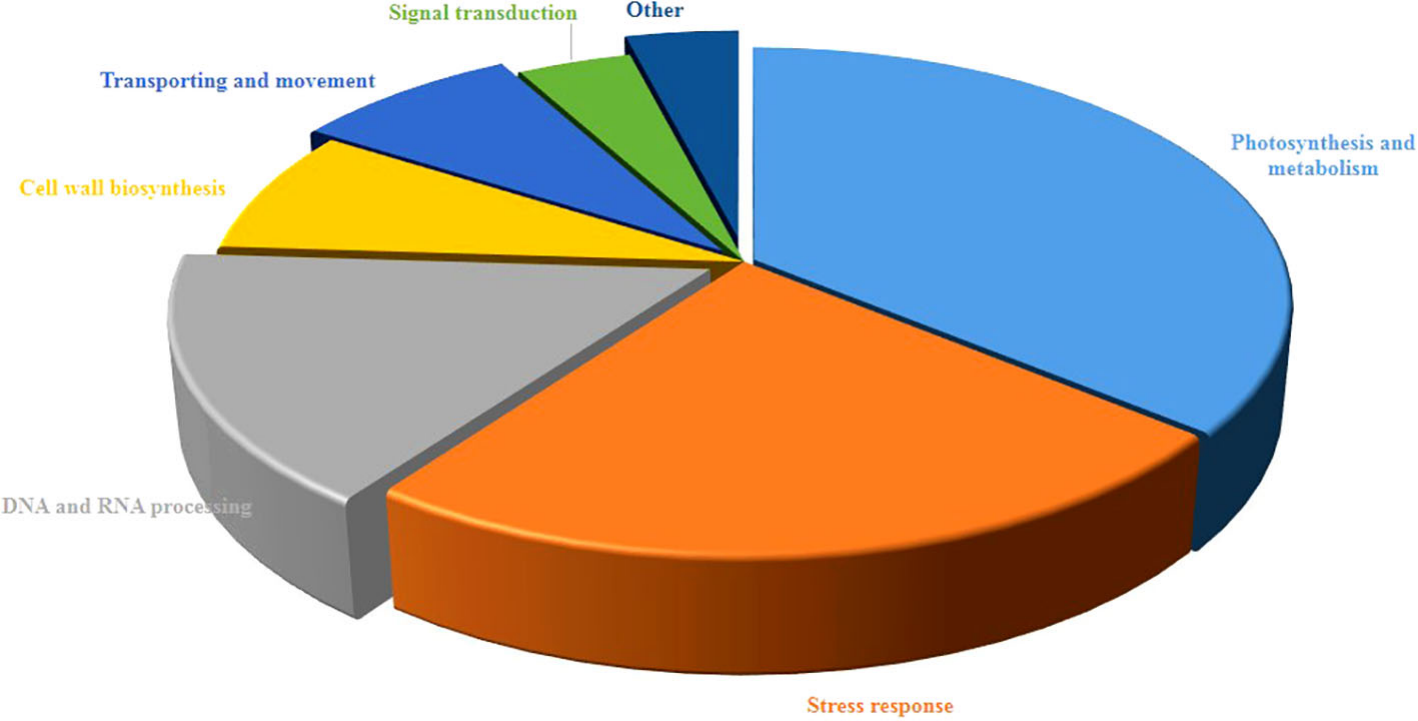
Functional classification of differentially regulated proteins of pistachio leaves under osmotic stress condition. The percentage of annotated proteins related to each pathway illustrated in pie charts.

As shown in **Figure 3**, the study of biological functions of differentially accumulated proteins led to their classification into seven diverse groups. Most of them contributed to photosynthesis and metabolism accounting for 36% then followed by stress response (24%), DNA and RNA processing (16%), and cell wall biosynthesis (8%), transporting (8%). Furthermore, the remaining proteins were associated with signal transduction (4%) and other (4%).

### Protein-chemical interaction

**Figure 4:**
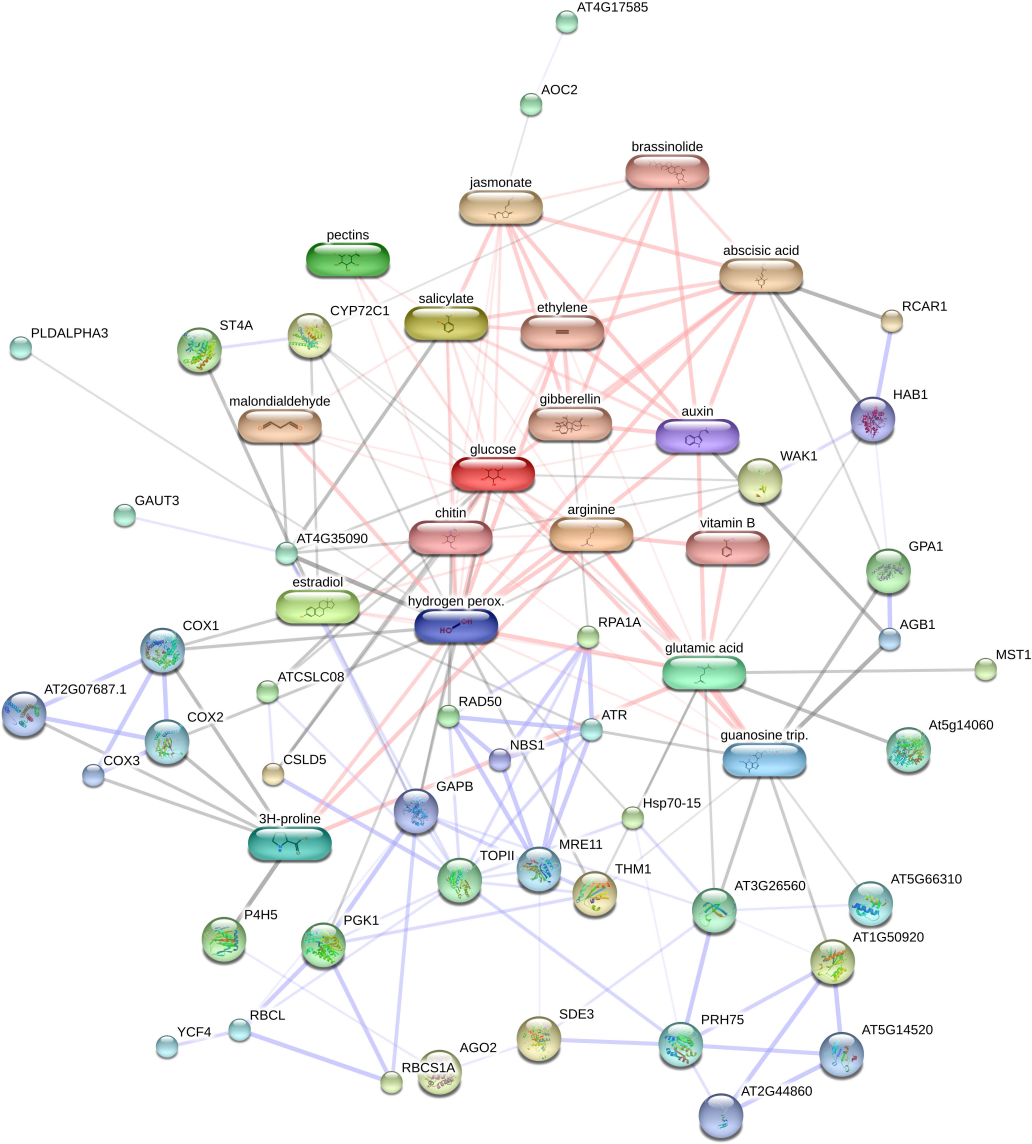
Analysis of the network of protein – chemical of identified proteins using STITCH 5.0. AT2G44860, large subunit ribosomal protein L24e; AT3G17530, F-box and associated interaction domain-containing protein; WAK1, Serine/threonine-protein kinase; At5g14060, aspartokinase 2; SDE3, SILENCINGDEFECTIVE; AT1G11780, alpha-ketoglutarate-dependent dioxygenase alkB; ST4A, sulfotransferase 4A; AGO2, argonaute 2;P4H5, prolyl 4-hydroxylase 5; AOC2, allene oxide cyclase 2; THM1, thioredoxin M1, RPA1A, replication factor A1; YCF4, unfolded protein binding; GPA1, G protein alpha subunit 1; NBS1, nijmegen breakage syndrome 1; PLDALPHA3, phospholipase D; RBCS1A, ribulose bisphosphate carboxylase small chain 1A, AT4G35090, catalase 2; TOPII, topoisomerase II; AT4G17585, aluminum activated malate transporter family protein;HAB1, HYPERSENSITIVE TO ABA1;CSLD5, 1,4-beta-D-xylan synthase; AT5G14520, pescadillo-related protein; MST1, thiosulfate sulfurtransferase;COX1, cytochrome oxidase; AT3G26560, ATP-dependent RNA helicase DHX8/PRP22; RCAR1, abscisic acid receptor PYL9; RAD50, DNA repair protein RAD50; RBCL, ribulose-bisphosphate carboxylases;ATCSLC08, putative xyloglucan glycosyltransferase 8; GAUT3, galacturonosyltransferase 3; PGK1, phosphoglycerate kinase 1;PRH75, DEAD-box ATP-dependent RNA helicase 7; AT5G66310, ATP binding microtubule motor family protein; ATR, serine/threonine-protein kinase ATR;AGB1, GTP binding protein beta 1; CYP72C1, cytochrome p450 72c1; AT2G07687.1, cytochrome c oxidase subunit 3; COX3, cytochrome c oxidase subunit 3;MRE11, MEIOTIC RECOMBINATION 11; COX2, cytochrome oxidase 2; Hsp70-15, Heat shock protein 70.

In this study, we evaluated the network of protein-protein/chemicals interactions involving in osmotic stress in pistachio leave using STITCH database against *Arabidopsis thaliana*. All of 25 identified proteins were detected with the STITCH database. The PCI network indicated a strong interaction network between identified proteins and several chemical compounds in different pathways (**Figure 4**). Identified chemical compounds related to plant response under osmotic stress were included proline, guanosine triphosphate, arginine, nicotinamide, H_2_O_2_, pectin, glucan, glutamate, phosphoglycerate kinase 1, phosphate, glucose, chitin, topoisomerase II, estradiol, malondialdehyde, cytochrome p450 72c1, cytochrome oxidase 2, cytochrome c oxidase subunit 3,1,4-beta-D-xylan synthase, allene oxide cyclase 2, putative nucleolar GTP-binding protein 1, ATP-dependent RNA helicase DHX8/PRP22, silencing defective, large subunit ribosomal protein L24e, cell wall-associated kinase, alpha-ketoglutarate-dependent dioxygenase alkB, ethylparaben, replication factor A1, G protein alpha subunit 1, magnesium chloride, hypersensitive to ABA1, pescadillo-related protein, putative xyloglucan glycosyltransferase 8, phosphoglycerate kinase 1. Also STITCH database were predicated that various pathways controlled by hormones and their crosstalk, consisting of brassinolide, gibberellin, ethylene, salicylic acid, ABA, auxin, and jasmonate.

### Gene ontology analysis

**Figure 5:**
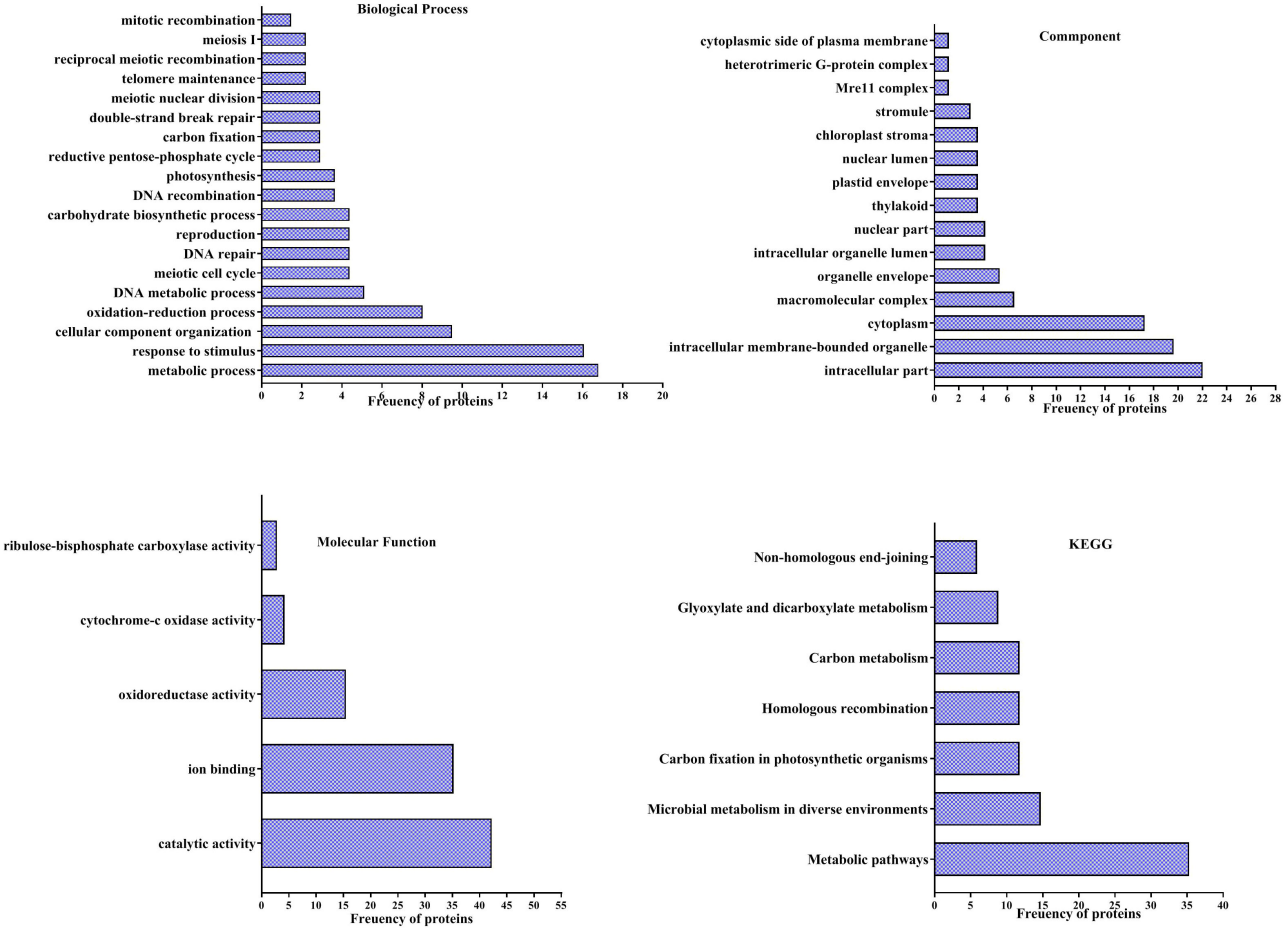
Analysis of Gene ontology (GO) of differentially accumulation proteins of pistachio leaves under osmotic condition. Pathways of four groups of functional enrichment consisting of biological process, cellular component, molecular function and KEGG were analyzed *via* the STITCH database.

According to the analyses of GO enrichment, investigated proteins were in various ranges of biological processes (**Figure 5**), included of metabolic process (16.78%), response to stimulus (16%), cellular component organization or biogenesis (9.48%), oxidation-reduction process (8%), DNA metabolic process (5.1%), meiotic cell cycle (4.38%), DNA repair (4.38%), reproduction (4.38%), carbohydrate biosynthetic process (4.38%), DNA recombination (3.65%), photosynthesis (3.65%), reductive pentose-phosphate cycle (2.2%), carbon fixation (2.2%), double-strand break repair (2.2%), meiotic nuclear division (2.2%), telomere maintenance (2.19%), reciprocal meiotic recombination (2.19%), meiosis I (2.19%) and mitotic recombination (1.46%).

Findings of the GO enrichment analyses displayed that identified proteins, under osmotic stress, mainly located in intracellular part (22%), intracellular membrane-bounded organelle (19.64%), cytoplasm (17.26%), macromolecular complex (6.54%), organelle envelope (5.35%), intracellular organelle lumen (4.16%), nuclear part (4.16%), thylakoid (3.57%), plastid envelope (3.75%), nuclear lumen (3.75%), chloroplast stroma (3.57%), stromule (2.97%), Mre11 complex (1.19%), heterotrimeric G-protein complex (1.19%) and cytoplasmic side of plasma membrane (1.19%) (**Figure 5**).

The enrichment of molecular functions illustrated the most processes related to catalytic activity (42.25%), ion binding (35.21%), oxidoreductase activity (15.49%), cytochrome-c oxidase activity (4.22%) and ribulose-bisphosphate carboxylase activity (2.81%) (**Figure 5**).

Evaluation of KEGG pathways demonstrated that differentially accumulated proteins enriched in metabolic pathways (35.29%), microbial metabolism in diverse environments (14.70%), Carbon fixation in photosynthetic organisms (11.76%), homologous recombination (11.76%), carbon metabolism (11.76%), glyoxylate and dicarboxylate metabolism (8.82%) and non-homologous end-joining (5.88%) (**Figure 5**).

### Analysis of transcriptional expression change by qRT-PCR

**Figure 6:**
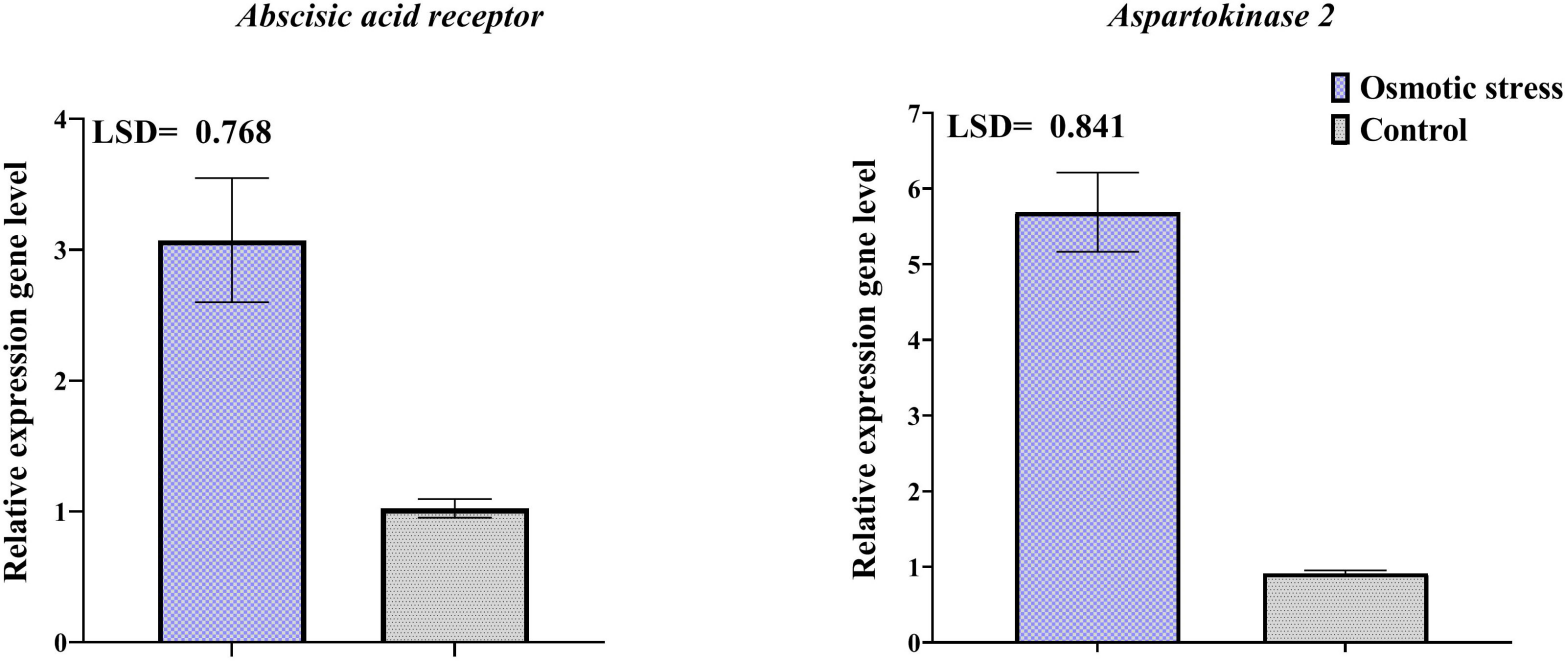
Transcriptional expression levels of two differentially abundant proteins in pistachio leaves in response to osmotic stress based on qRT-PCR analysis results. Error bars indicated the SD (n = 3) Statistic were carried out at p = 0.05 according to the LSD test.

Changes in the transcription level of three selected genes of differentially abundant proteins were investigated by qRT-PCR (**Figure 6**). Results of qRT-PCR analysis indicated that transcription level of genes related to spots 116 and 133 increased in response to osmotic stress. The change of the expression level of representative genes was the same as their protein expression.

## Discussion

Plants have established numerous defense mechanisms against osmotic stress such as osmotic adjustment by ion transport reregulation and osmoprotectants synthesis, preservation of membrane stability, activation of antioxidant defense, reregulation of cell cycle, and metabolic changes (Xiong and Zhu, 2002; Zhang and Shi, 2018). Polyethylene glycol (PEG), which has no toxic effect on the plant, induces osmotic stress by withdrawing water from the protoplasm and apoplast (Toorchi et al., 2009). In this study, the altered contents of some biochemical compounds and several differences in protein expression patterns due to dehydration resulted from PEG were detected in pistachio.

### Biochemical Parameters

Tolerant plants to osmotic stress have potency to sustain homeostasis of metabolic using increase of different solutes (Blum, 2017). In this study, accumulation of several organic solutes like proline and phenolics were increased, indicating a positive role of these compounds in the pistachio plant under osmotic stress. Proline acts not only as a compatible osmolyte but also as a ROS scavenger, a buffer for cellular redox potential, and a nutritional source under stress (Hayat et al., 2012; Chun et al., 2018). An increase of about six-fold in proline content was observed here. An increase in proline accumulation as an osmolyte in response to dehydration has been observed in a wide range of plants (Skirycz et al., 2010; Benhassaini et al., 2012; Kim et al., 2016; Jungklang et al., 2017; Zegaoui et al., 2017; Koenigshofer and Loeppert, 2019; Mattioli et al., 2020). Pálet al. (Pál et al., 2018) had also confirmed the enhancement in the amount of proline under osmotic stress. Owing to ability to forgive hydrogen, decrease and extinguish radical oxygen, phenolics have oxidation virtues and have a main role as sweepers of ROS in plant under various stresses (Naikoo et al., 2019; Mechri et al., 2020). A potent relation exists among osmotic tolerance and increased accumulation of phenolic compounds (Dey and Bhattacharjee, 2020; Naikoo et al., 2019). Piwowarczyk et al. (Piwowarczyk et al., 2017) reported that concentration of phenols elevated in grass pea plant under PEG-induced osmotic stress, similar to our results. Several studies illustrated that concentration of proline and phenols increased under salinity and drought stress in pistachio plants (Khoyerdi et al., 2016; Akbari et al., 2018; Jamshidi Goharrizi et al., 2020). Therefore, considering the presented results and literature data, the increased accumulation of phenolics and proline may be propounded as a main elements related to the tolerance of pistachio to osmotic stress.

Lipid peroxidation of cell leading to produce malondialdehyde indicating severity of injury to the cell membrane (Morales and Munné-Bosch, 2019). Our data showed that malondialdehyde content did not changed that maybe due to the physiological adaptation and or elevated activity of antioxidant systems that diminished ROS levels and membrane injury. On the other hand, our result contradicts results of Khoyerdi et al. (Khoyerdi et al., 2016) and Goharrizi et al. (Goharrizi et al., 2020). The principal reason for this conflict is probably differences in type of reaction pistachio varieties to osmotic stress as well as differences in the way of implementing stress treatment.

The photosynthesis process and its severity rely on content of pigments such as Chl a, Chl b, and carotenoids and effect the biological productivity. Also, photosynthetic pigments harvest the light for photosynthesis process (Rahneshan et al., 2018; Lan et al., 2020). Osmotic condition can injury the chlorophyll content and prevent synthesis of chlorophyll pigments, therefore chlorophyll degradation is one of the subsequences of osmotic stress (Akbari et al., 2018; García-Morales et al., 2018). In this study, decline in chlorophyll content were observed under osmotic stress. In general, this reduction can be imputed to various factors, such as the sluggish synthesis or rapid degradation of the pigments in cells, decrease in synthesis of chlorophylls, derangement in the complex of pigment–protein and thought deficits in ions that are necessary for chlorophyll biosynthesis (Akbari et al., 2018; Behzadi Rad et al., 2021; Zhu et al., 2021). Lan et al. (Lan et al., 2020) reported that chlorophyll content remarkably reduced in wheat under PEG-induced osmotic stress, similar to our results. Also, our findings are in compliance with (Behzadi Rad et al., 2021) who reported that the chlorophyll content of pistachio leaves reduce under salinity condition. Carotenoids function as photoprotection by absorbing extreme light and protect chloroplasts from harmful ROS level therefore protect chlorophyll from major damage (Rahneshan et al., 2018). In this study, no change in carotenoids concentration was observed (**Figure 1**).

### Photosynthesis and metabolism related proteins

The most important enzyme in photosynthesis is ribulose bisphosphate carboxylase (Rubisco), which consists of two types of large and small subunits, plays main role in the fixation of CO_2_.

The alteration in photosynthesis of plants highly related to the Rubisco activity. It has been reported that drought stress had a harmful impact on the function of Rubisco in various plants, led to decrease of biosynthesis and degradation of subunits and finally diminution of photosynthesis (Zhang et al., 2016; Shayan et al., 2020). In this study, subunits of Rubisco down-regulated which was agreement with our pervious study (Pakzad et al., 2019). Also, Jamshidi Goharrizi et al. (Jamshidi Goharrizi et al., 2020) using analyzing the leaf proteome profiling of pistachio demonstrated that ribulose bisphosphate carboxylase/oxygenase large chains were down-regulated under salinity stress condition.

OEE1 is one of member of the PSII related to photoreactions, and plays a role in stabilizing the cluster of Mn in the PSII that is initial locate of water splitting. A loss of this protein leads to a full inability for evolve oxygen in PSII (Chaves et al., 2009; Dubey, 2018; Heide et al., 2004; Mayfield, 1999). The decreased levels of this protein were occurred under osmotic stress. In contrast to our results Buendig et al. (Buendig et al., 2016), reported a decrease in the abundance of OEE1 in potato genotypes under osmotic stress. A diminish in the accumulation of OEE1 maybe therefore due to harm to PSII and represents a considerable decrease of efficiency photosynthesis under osmotic stress (Chaves et al., 2009; Dubey, 2018). PSI assembly protein Ycf4 (YCF4) plays a significant role in the assembly of PSI and its firm hold to the thylakoid membrane (Nellaepalli et al., 2018; Hui-Hui et al., 2019). In this study, we found that the accumulation of YCF4 were reduced under osmotic stress, illustrating that osmotic stress reduce quantity and integrity of PSI protein. Cytochrome c oxidase subunit II PS17 is the final enzyme related to respiratory chain, oxidizing cytochrome c and make molecular water using transfer electrons to molecular oxygen. It has been reported that alterations in expression of the cytochrome c oxidase level were attended with alterations in the accumulation of proteins related to photosynthesis and carbohydrate metabolism under stress condition (Çulha Erdal et al., 2021). Increased accumulation of this protein has been reported by Çevik et al. (Çevik et al., 2019) and Çulha Erdal et al. (Çulha Erdal et al., 2021) under drought stress. Abundance of this protein was elevated by osmotic stress in pistachio, indicating that maybe help to generation of energy *via* respiratory chain, leading to improve photosynthesis and carbohydrate metabolism levels. Totally, the decrease in the expression of OEE1, Ycf4, and ribulose bisphosphate carboxylase, implicitly indicated destructive effect of osmotic stress on the photosynthesis process.

Glyceraldehyde-3-phosphate dehydrogenase (GAPDH) plays a vital role in the physiological plant function and energy production *via* glycolytic pathway and protect of photosystem II from ROS under stress condition (Fermani, 2007; Bertomeu et al., 2010). the study conducted by Kappachery et al. (Kappachery et al., 2021) indicated that Overexpression of gene encoding GAPDH in *Arabidopsis thaliana* transgenic elevate antioxidant enzymes, photosynthetic pigments and improve photosynthesis *via* increasing general PSII efficiency under salt stress. In our study, GAPDH accumulation elevated in pistachio leaves treated with PEG6000, revealing that this enzyme may provide the way for obtaining extra energy for regulation of cellular homeostasis and also it maintain photosynthetic efficiency using protect photosystem II from adverse effect of osmotic stress.

Aspartate kinase (AK) is the primary and the most vital enzyme in phosphorylating L-aspartate, leading to biosynthesizing four necessary amino acids: methionine, threonine, lysine, and isoleucine (Jander and Joshi, 2009; Han et al., 2021). There are several evidences demonstrated AK involved in osmotic stress (Chefdor et al., 2006; Héricourt et al., 2013; Héricourt et al., 2016) and drought and nutritional stress (Curtis et al., 2018). In this study the increased accumulation of AK was determined, suggesting biosynthesis of numerous amino acid leading to improve plant adaption abilities under osmotic stress.

The function of the sulfotransferase superfamily is to transfer a sulfuryl group from the general donor, PAPS, to a hydroxyl group from a wide range of substrates, including glucosinolates, phenolic acids, flavonoids, brassinosteroids, coumarins, jasmonates, and terpenoids. They are involved in very diverse physiological functions such as a response to pathogen or detoxification (Hernàndez-Sebastiá et al., 2008; Hirschmann et al., 2014). In this study two proteins belong to sulfotransferase superfamily; Thiosulfate/3-mercaptopyruvate sulfurtransferase 1and sulfotransferase 4 was found to be increased under osmotic stress. It has been reported that sulfurtransferases play a main role in ROS, cyanide, and heavy metals detoxification and contribute to metabolism of sulfur and cysteine (Papenbrock and Schmidt, 2000; Nakamura et al., 2000; Most and Papenbrock, 2015; Yamasaki and Cohen, 2016). Proteomic profiling on lettuce was conducted by Leitão et al. (Leitão et al., 2021) indicated that accumulation of sulfurtransferase was increased under stress induced by pharmaceutical contamination. Also, several studied illustrated that sulfurtransferases play a main role in plant response to abiotic stress such as osmotic, salt and hormone stress (Jin et al., 2019). Overall, increased expression of cytosolic sulfotransferase 4 and thiosulfate/3-mercaptopyruvate sulfurtransferase 1 indicated a wide range of changes associated with osmotic stress.

### Stress response related proteins

Phospholipase D is a most important enzyme involved in hydrolyzing membrane phospholipids, leading to generate phosphatidic acid which act as a signaling molecule so that promotes stomatal closure under osmotic stress (Saucedo-García et al., 2015; Rodas-Junco et al., 2021). Many evidences illustrate that PLD has an important role in plant tolerance under stress (Ji et al., 2018; Alferez et al., 2019; Gnanaraj et al., 2021; Wei et al., 2022) and adjust plant defense response to osmotic stress (Liu et al., 2021; Liu et al., 2022; Hong et al., 2008). Our findings indicated that abundance of Phospholipase D was increased under osmotic stress, similar to results of (Urban et al., 2021).

Osmotic stress induce the ABA level in various plants, which is a well-known reality (Haider et al., 2018; Kai etal., 2019; Xing et al., 2016). In this study, the up-regulation of abscisic acid receptor PYL9 was observed, indicating activation of pathway related to ABA signaling in pistachio plant toward response to osmotic stress. Transcriptomic analysis of grapevine leaves indicated that PYL9 induced under salt stress (Guan et al., 2018) Also, it Miao et al. (Miao et al., 2018) reported that PYL9 overexpression improves drought resistance.

Up-regulation HSPs, as chaperones, involved in facilitating protein conformation or refolding under stress conditions, because, denaturation of proteins occurs as a result of the reduction of water content in osmotic stress (Zang and Komatsu, 2007). The expressions of HSP genes are prompted by denatured or damaged proteins (Xiong and Zhu, 2002).Thus, their expressions are up-regulated in the stress or some stages of growth and development (Park and Seo, 2015).The elevated expression of a heat shock protein under osmotic stress was observed in the present study. This result is agreement to studies of Rahman et al. (Rahman et al., 2015) who reported increased accumulation of 18.1 kDa class I heat shock protein and other HSPs in transgenic sugarcane under drought stress induced with PEG.

Following many stresses, oxidative stress also occurs due to augmented ROS. Although ROS plays a key role in signaling and regulation of many genes (Xiong and Zhu, 2002), enhancing its concentration damages cellular structures seriously, so some mechanisms have been established in the plant to prevent the overproduction or to remove ROS (Toorchi et al., 2009), including catalase activation or biosynthesis, which sweeps away H_2_O_2_ by its activity. Increasing antioxidants as a common result of most abiotic stresses, improves plant stress tolerance. Abiotic stress induces genes for various catalase isoforms (Xiong and Zhu, 2002). Catalase in interaction with plant natriuretic peptide (PNP) triggers the regulation of ROS levels and cell redox homeostasis during the salinity or drought stress (Turek et al., 2020). In this study two spots (119 and 173) identified as catalase enzyme that their expression were increased under osmotic stress. Augmented activity of the antioxidant system due to increased expression of relevant proteins during osmotic stress has been observed in other studies, as well (Toorchi et al., 2009; Zhang and Shi, 2018).

Serine/threonine-protein kinase CST is a receptor-like cytoplasmic kinase that acts as an inhibitor in such a way that limits the extent of cell separation signaling, and causes cells to be separated only in designated areas in abscission zone (Burr et al., 2011). Several researches have been proven positive role of Serine/threonine-protein kinases under stress condition in different plants (Mao et al., 2010; Sun et al., 2013; Rampino et al., 2017; Mao et al., 2010). In this study, activation of Serine/threonine-protein kinase CST was increased. The role of this protein in response plant to biotic stress was proved (Ghorbani et al., 2019).

### DNA and RNA processing related proteins

Plants to dominate the stable challenge from a swiftly altering environment have several particular adaptation mechanisms, among which DNA and RNA processing are main strategies (Wong et al., 2017; Song et al., 2021). In this study several proteins related to DNA and RNA processing were identified included of DEAD-box ATP-dependent RNA helicase 7, DNA repair protein RAD50, replication protein A 70 kDa DNA-binding subunit D, and argonaute MEL1 are proteins that participate in the processes assigned to DNA and RNA (Nonomura et al., 2007; Takashi et al., 2009; Gherbi et al., 2001; Gallego et al., 2001; Aubourg et al., 1999). This display that plant to adapt under osmotic condition could increase several transcriptional and translation processes and seriously elevated the stability and variety of proteins (Aubourgrt al., 1999; Nonomura et al., 2007). The role of DNA and RNA processing has been indicated in studies related to various environmental stress (Gao et al., 2019; Li et al., 2020; Marondedze et al., 2020)

### Cell wall biosynthesis related proteins

Like other results (Ngara et al., 2018; Zhang and Shi, 2018; Wang et al., 2020), we also recognized some proteins associated with cell wall construction, including prolyl 4-hydroxylase 5 and probable galacturonosyltransferase 3, since the cell wall is the protective barrier and the first front of defense against stress. Cell division, which requires the formation of a new cell wall, is also inhibited under osmotic stress (Xiong and Zhu, 2002). Hence, cell metabolism changes to guide plant status from optimal growth to stress-adapted growth, which requires alterations in the expression and the activity of many proteins assigned to the intercellular space and cell wall (Ngara et al., 2018). The synthesis of pectin, which is a component of the cell wall, requires the activity of galacturonosyltransferase (Boustani et al., 2017). 4-hydroxyproline, which is an important component of cell wall glycoproteins, is produced post-translationally by the activity of Prolyl 4-hydroxylase 5 on proline-rich sequences in glycoproteins (Velasquez et al., 2011). In this study proteins related to cell wall stabilization was increased, indicating that these proteins might help to the more consolidation of cell wall in pistachio and leads to more osmotic tolerance and plant could regulate the osmotic potential using changes to the cell wall.

### Transporting and movement related proteins

Kinesin-like proteins are motors that perform microtubule-based movement, such as the transport of vesicles and organs, chromosome segregation, and signal transduction, thus play a key role in developmental and environmental processes (Ni et al., 2005). Changes in the expression of microtubule-related proteins may alter the morphology of stressed tissue. In this research, kinesin-like protein KIN-7H revealed higher abundance in the PEG treatment.

Aluminum-activated malate transporter, which belongs to the anion channels, is located in the membranes of different tissues and has a various and fundamental range of physiological functions such as aluminum resistance, signaling, anion homoeostasis, osmotic adjustment, stomata regulation, and abiotic stress tolerance *via* transporting malate or inorganic anions (Palmer et al., 2016; Ramesh et al., 2018; Hejri et al., 2021). Maintaining sufficient amounts of water is essential for plant growth and development (Pasaribu et al., 2021). Therefore, the plant controls the ionic balance in the cell to regulate the osmotic pressure during the osmotic stress. Thus, aluminum-activated malate transporter 11 can play a significant role in this process by transporting malate and inorganic anions. Scientific reports suggest that abiotic stresses such as salinity regulate transporters either at the protein or mRNA levels (Xiong and Zhu, 2002).

### Signal transduction related protein

Under osmotic stress, plants sense the stress through signal transmission networks and as a result start to react. They adapt to stress conditions through different signaling pathways that affect a wide range of protein expression (Zandalinas et al., 2020). Alterations were reported in levels of various proteins related to signal transduction under stress condition (Shan et al., 2018; Meena et al., 2019; Zhang et al., 2019; Wang et al., 2022). A main protein to take a notice is guanine nucleotide-binding proteins, named G proteins or GTPases. These proteins *via* activity of moderator or transducers in different signaling systems located in transmembrane adjust many cellular processes such as secretion, transport etc. (Patel et al., 2020). G-proteins consisting of the Gα, Gβ, and Gγ subunits, Based on our findings, The Gα subunit up-regulated under osmotic stress. Gα subunit is the vital the member of G-protein signal transduction so that activation of G-protein and downstream signal depended on Gα (Liu et al., 2021; Liu et al., 2018; Pandey and Assmann, 2004). Similar to our findings, several studies illustrated that increased expression of the Gα subunit plays a significant role in plant resistance to various abiotic stress such as salt (Misra et al., 2007), drought (Ferrero-Serrano and Assmann, 2016), heat and cold (Chakraborty et al., 2015; Chakraborty et al., 2015; Ma et al., 2015; Guo et al., 2020).

### Other protein

F-box kelch-repeat proteins can adjust biosynthesis of phenylpropanoid *via* regulating the turnover of phenylalanine ammonia-lyase and also play a main role in a main role in providing homeostasis *via* eliminating misfolded or injured proteins which could destroy cellular activations (Zhang et al., 2013; Kamireddy et al., 2021). In this study, the expression level of the F-box/Kelch-repeat protein (At3g17530) was elevated under stress. To date, the function of this protein is unclear but several studies indicated that proteins belong to F-box/Kelch-repeat protein family play an important role in improve of tolerance plant under stress condition (Curtis et al., 2013; Wang et al., 2017; Venkatesh et al., 2020).

### STITCH and GO analysis

Protein–protein/chemical interactions can notably modulate different cellular activities, such as replication, transcriptional regulation, defense responses, growth and development, processes of signaling, and consonance of numerous metabolic pathways (Fukao, 2012; Braun et al., 2013). In this study, the interaction networks proteins-proteins/chemicals in pistachio leaves treated by PEG were analyzed using STITCH. The STITCH network predicted 33 proteins and small molecules interacted with identified proteins using proteomic technic. Also STITCH analysis indicated that all of proteins and small molecules regulation by a set of hormones and their crosstalk.

According to KEGG analysis, the most proteins involved in response to osmotic in pistachio leaves enriched in metabolic pathways that was similar to proteomic findings, indicating the osmotic stress mostly impacts the metabolic pathways.

## Conclusion

To better our knowledge about plant tolerance under osmotic stress and molecular mechanisms behind related responses, proteomics of pistachio leaves was performed. Osmotic stress imposed a change in the expression of 25 proteins. 21 proteins were highly expressed while four proteins were less expressed. These identified proteins function in several biological processes such as stress response, photosynthesis and metabolism, DNA and RNA processing, and cell wall biosynthesis which point out their roles in adaptation of pistachio under osmotic stress. Based on KEGG analysis, proteins related to metabolic pathways have the most vital role in pistachio response to osmotic stress. The decline in the expression of Rubisco, OEE1, and photosystem I assembly protein Ycf4 suggested the destructive effect of membrane dehydration resulted from osmotic stress on the photosynthesis process. Altered expression of some proteins associated with the cell wall was expected because the wall is the first defense barrier against stress, and cell division, which requires the formation of a new wall, is inhibited under stress as well. Some proteins involved in DNA and RNA processing were also overexpressed because osmotic stress activates signaling pathways such as the ABA-related pathway, which ultimately leads to altered gene expression and delayed cell division, and stress-induced ROS may also damage DNA.

## Data availability statement

The datasets presented in this study can be found in online repositories. The names of the repository/repositories and accession number(s) can be found in the article/supplementary material.

## Author contributions

RP performed the experiments, analyzed the data and wrote the original draft. FF conceived and designed the study, supervised all steps, review and edit the manuscript, approved the final version to be published. MK investigate and MM analyzed the data, review and edit the manuscript.

## References

[B14] Ainsworth E. A. Gillespie K. M. (2007). Estimation of total phenolic content and other oxidation substrates in plant tissues using folin–ciocalteu reagent. Nat. Protoc. 2 (4), 875–877. doi: 10.1038/nprot.2007.102 17446889

[B39] Akbari M. Mahna N. Ramesh K. Bandehagh A. Mazzuca S. (2018). Ion homeostasis, osmoregulation, and physiological changes in the roots and leaves of pistachio rootstocks in response to salinity. Protoplasma 255 (5), 1349–1362. doi: 10.1007/s00709-018-1235-z 29527645

[B86] Alferez F. Wu J. Graham J. H. (2019). Phospholipase d (PLD) response to water stress in citrus roots and leaves. Agronomy 10 (1), 45. doi: 10.3390/agronomy10010045

[B113] Aubourg S. Kreis M. Lecharny A. (1999). The DEAD box RNA helicase family in arabidopsis thaliana. Nucleic Acids Res. 27 (2), 628–636. doi: 10.1093/nar/27.2.628 9862990 PMC148225

[B47] Behzadi Rad P. Roozban M. R. Karimi S. Ghahremani R. Vahdati K. (2021). Osmolyte accumulation and sodium compartmentation has a key role in salinity tolerance of pistachios rootstocks. Agriculture 11 (8), 708. doi: 10.3390/agriculture11080708

[B11] Benhassaini H. Fetati A. Hocine A. K. Belkhodja M. (2012). Effect of salt stress on growth and accumulation of proline and soluble sugars on plantlets of pistacia atlantica desf. subsp. atlantica used as rootstocks. BASE. 16 (2), 159–165.

[B26] Blum A. (2017). Osmotic adjustment is a prime drought stress adaptive engine in support of plant production. Plant Cell environment. 40 (1), 4–10. doi: 10.1111/pce.12800 27417527

[B19] Blum H. Beier H. Gross H. J. (1987). Improved silver staining of plant proteins, RNA and DNA in polyacrylamide gels. Electrophoresis 8 (2), 93–99. doi: 10.1002/elps.1150080203

[B53] Boustani A. Fatehi F. Azizinezhad R. (2017). The proteome response of “Hordeum marinum” to long-term salinity stress. Cereal Res. Commun. 45 (3), 401–410. doi: 10.1556/0806.45.2017.020

[B18] Bradford M. M. (1976). A rapid and sensitive method for the quantitation of microgram quantities of protein utilizing the principle of protein-dye binding. Analytical Biochem. 72 (1-2), 248–254. doi: 10.1016/0003-2697(76)90527-3 942051

[B140] Braun P. Aubourg S. Van Leene J. De Jaeger G. Lurin C. (2013). Plant protein interactomes. Annu. Rev. Plant Biol. 64, 161–187. doi: 10.1146/annurev-arplant-050312-120140 23330791

[B58] Buendig C. Jozefowicz A. M. Mock H.-P. Winkelmann T. (2016). Proteomic analysis of two divergently responding potato genotypes (Solanum tuberosum l.) following osmotic stress treatment *in vitro* . J. Proteomics 143, 227–241. doi: 10.1016/j.jprot.2016.04.048 27153758

[B103] Burr C. A. Leslie M. E. Orlowski S. K. Chen I. Wright C. E. Daniels M. J.. (2011). CAST AWAY, a membrane-associated receptor-like kinase, inhibits organ abscission in arabidopsis. Plant Physiol. 156 (4), 1837–1850. doi: 10.1104/pp.111.175224 21628627 PMC3149937

[B13] Carillo P. Gibon Y. (2011). Protocol: extraction and determination of proline. PrometheusWiki 2011, 1–5.

[B63] Çevik S. Akpinar G. Yildizli A. Kasap M. Karaosmanoğlu K. Ünyayar S. (2019). Comparative physiological and leaf proteome analysis between drought-tolerant chickpea cicer reticulatum and drought-sensitive chickpea c. arietinum. J. biosciences. 44 (1), 1–13. doi: 10.1007/s12038-018-9836-4 30837371

[B132] Chakraborty N. Sharma P. Kanyuka K. Pathak R. R. Choudhury D. Hooley R.. (2015). G-Protein α-subunit (GPA1) regulates stress, nitrate and phosphate response, flavonoid biosynthesis, fruit/seed development and substantially shares GCR1 regulation in a. thaliana. Plant Mol. Biol. 89 (6), 559–576. doi: 10.1007/s11103-015-0374-2 26346778

[B133] Chakraborty N. Singh N. Kaur K. Raghuram N. (2015). G-Protein signaling components GCR1 and GPA1 mediate responses to multiple abiotic stresses in arabidopsis. Front. Plant science. 6, 1000. doi: 10.3389/fpls.2015.01000 PMC4649046 26635828

[B56] Chaves M. Flexas J. Pinheiro C. (2009). Photosynthesis under drought and salt stress: regulation mechanisms from whole plant to cell. Ann. botany. 103 (4), 551–560. doi: 10.1093/aob/mcn125 18662937 PMC2707345

[B69] Chefdor F. Bénédetti H. Depierreux C. Delmotte F. Morabito D. Carpin S. (2006). Osmotic stress sensing in populus: components identification of a phosphorelay system. FEBS letters. 580 (1), 77–81. doi: 10.1016/j.febslet.2005.11.051 16359674

[B28] Chun S. C. Paramasivan M. Chandrasekaran M. (2018). Proline accumulation influenced by osmotic stress in arbuscular mycorrhizal symbiotic plants. Front. Microbiol. 9, 2525. doi: 10.3389/fmicb.2018.02525 30459731 PMC6232873

[B62] Çulha Erdal Ş Eyidoğan F. Ekmekçi Y. (2021). Comparative physiological and proteomic analysis of cultivated and wild safflower response to drought stress and re-watering. Physiol. Mol. Biol. Plants. 27 (2), 281–295. doi: 10.1007/s12298-021-00934-2 33707869 PMC7907392

[B72] Curtis T. Y. Bo V. Tucker A. Halford N. G. (2018). Construction of a network describing asparagine metabolism in plants and its application to the identification of genes affecting asparagine metabolism in wheat under drought and nutritional stress. Food Energy security. 7 (1), e00126. doi: 10.1002/fes3.126 29938110 PMC5993343

[B137] Curtis R. H. Powers S. J. Napier J. Matthes M. C. (2013). The arabidopsis f-box/Kelch-repeat protein At2g44130 is upregulated in giant cells and promotes nematode susceptibility. Mol. Plant-Microbe interactions. 26 (1), 36–43. doi: 10.1094/MPMI-05-12-0135-FI 23075039

[B41] Dey N. Bhattacharjee S. (2020). Accumulation of polyphenolic compounds and osmolytes under dehydration stress and their implication in redox regulation in four indigenous aromatic rice cultivars. Rice Science. 27 (4), 329–344. doi: 10.1016/j.rsci.2020.05.008

[B57] Dubey R. S. (2018). “Photosynthesis in plants under stressful conditions,” in Handbook of photosynthesis (Boca Raton: CRC Press), 629–649.

[B8] Esmaeilpour A. Van Labeke M.-C. Samson R. Van Damme P. (2015). Osmotic stress affects physiological responses and growth characteristics of three pistachio cultivars. Acta Physiologiae Plantarum. 37 (6), 1–14. doi: 10.1007/s11738-015-1876-x

[B9] FAO (2020). “FaAOotUN,” in FAOSTAT, United Nations. vol. 2020.

[B20] Fatehi F. Hosseinzadeh A. Alizadeh H. Brimavandi T. (2013). The proteome response of hordeum spontaneum to salinity stress. Cereal Res. Commun. 41 (1), 78–87. doi: 10.1556/CRC.2012.0017

[B21] Fatehi F. Hosseinzadeh A. Alizadeh H. Brimavandi T. Struik P. C. (2012). The proteome response of salt-resistant and salt-sensitive barley genotypes to long-term salinity stress. Mol. Biol. Rep. 39 (5), 6387–6397. doi: 10.1007/s11033-012-1460-z 22297690

[B65] Fermani S. Sparla F. Falini G. Martelli P. Casadio R. Pupillo P.. (2007). Molecular mechanism of thioredoxin regulation in photosynthetic A2B2-glyceraldehyde-3-phosphate dehydrogenase. Proc. Natl. Acad. Sci. 104 (26), 11109–11114. doi: 10.1073/pnas.0611636104 17573533 PMC1904167

[B131] Ferrero-Serrano Á Assmann S. M. (2016). The α-subunit of the rice heterotrimeric G protein, RGA1, regulates drought tolerance during the vegetative phase in the dwarf rice mutant d1. J. Exp. botany. 67 (11), 3433–3443. doi: 10.1093/jxb/erw183 27194741 PMC4892740

[B141] Fukao Y. (2012). Protein–protein interactions in plants. Plant Cell Physiol. 53 (4), 617–625. doi: 10.1093/pcp/pcs026 22383626

[B114] Gallego M. E. White C. I. (2001). RAD50 function is essential for telomere maintenance in arabidopsis. Proc. Natl. Acad. Sci. 98 (4), 1711–1716. doi: 10.1073/pnas.98.4.1711 11172016 PMC29322

[B112] Gao Y. Cui Y. Long R. Sun Y. Zhang T. Yang Q.. (2019). Salt-stress induced proteomic changes of two contrasting alfalfa cultivars during germination stage. J. Sci. Food Agriculture. 99 (3), 1384–1396. doi: 10.1002/jsfa.9331 30144052

[B44] García-Morales S. Gómez-Merino F. Trejo-Téllez L. Tavitas-Fuentes L. Hernández-Aragón L. (2018). Osmotic stress affects growth, content of chlorophyll, abscisic acid, na+, and k+, and expression of novel NAC genes in contrasting rice cultivars. Biol. plantarum. 62 (2), 307–317. doi: 10.1007/s10535-017-0761-4

[B115] Gherbi H. Gallego M. E. Jalut N. Lucht J. M. Hohn B. White C. I. (2001). Homologous recombination in planta is stimulated in the absence of Rad50. EMBO Rep. 2 (4), 287–291. doi: 10.1093/embo-reports/kve069 11306548 PMC1083866

[B107] Ghorbani A. Tahmasebi A. Izadpanah K. Afsharifar A. Dietzgen R. G. (2019). Genome-wide analysis of alternative splicing in zea mays during maize Iranian mosaic virus infection. Plant Mol. Biol. Reporter. 37 (5), 413–420. doi: 10.1007/s11105-019-01169-y

[B88] Gnanaraj M. Baburajan R. Sekar T. Muneewaran T. Manoharan K. (2021). Isolation and characterization of phospholipase d in response to abiotic stress from vigna radiata (L.) wilczek. Plant Gene 27, 100308. doi: 10.1016/j.plgene.2021.100308

[B43] Goharrizi K. J. Baghizadeh A. Kalantar M. Fatehi F. (2020). Combined effects of salinity and drought on physiological and biochemical characteristics of pistachio rootstocks. Scientia Horticulturae. 261, 108970. doi: 10.1016/j.scienta.2019.108970

[B95] Guan L. Haider M. S. Khan N. Nasim M. Jiu S. Fiaz M.. (2018). Transcriptome sequence analysis elaborates a complex defensive mechanism of grapevine (Vitis vinifera l.) in response to salt stress. Int. J. Mol. Sci. 19 (12), 4019. doi: 10.3390/ijms19124019 30545146 PMC6321183

[B98] Guo X. Li J. Zhang L. Zhang Z. He P. Wang W.. (2020). Heterotrimeric G-protein α subunit (LeGPA1) confers cold stress tolerance to processing tomato plants (Lycopersicon esculentum mill). BMC Plant Biol. 20 (1), 1–16. doi: 10.1186/s12870-020-02615-w 32847511 PMC7448358

[B94] Haider M. S. Kurjogi M. M. Khalil-ur-Rehman M. Pervez T. Songtao J. Fiaz M.. (2018). Drought stress revealed physiological, biochemical and gene-expressional variations in ‘Yoshihime’peach (Prunus persica l) cultivar. J. Plant Interactions. 13 (1), 83–90. doi: 10.1080/17429145.2018.1432772

[B68] Han M. Zhang C. Suglo P. Sun S. Wang M. Su T. (2021). L-aspartate: An essential metabolite for plant growth and stress acclimation. Molecules 26 (7), 1887. doi: 10.3390/molecules26071887 33810495 PMC8037285

[B27] Hayat S. Hayat Q. Alyemeni M. N. Wani A. S. Pichtel J. Ahmad A. (2012). Role of proline under changing environments: a review. Plant Signaling behavior. 7 (11), 1456–1466. doi: 10.4161/psb.21949 22951402 PMC3548871

[B55] Heide H. Kalisz H. M. Follmann H. (2004). The oxygen evolving enhancer protein 1 (OEE) of photosystem II in green algae exhibits thioredoxin activity. J. Plant Physiol. 161 (2), 139–149. doi: 10.1078/0176-1617-01033 15022827

[B122] Hejri S. Salimi A. Malboobi M. A. Fatehi F. (2021). Comparative proteome analyses of rhizomania resistant transgenic sugar beets based on RNA silencing mechanism. GM Crops Food. 12 (1), 419–433. doi: 10.1080/21645698.2021.1954467 34494497 PMC8820250

[B70] Héricourt F. Chefdor F. Bertheau L. Tanigawa M. Maeda T. Guirimand G.. (2013). Characterization of histidine-aspartate kinase HK1 and identification of histidine phosphotransfer proteins as potential partners in a populus multistep phosphorelay. Physiologia Plantarum. 149 (2), 188–199. doi: 10.1111/ppl.12024 23330606

[B71] Héricourt F. Chefdor F. Djeghdir I. Larcher M. Lafontaine F. Courdavault V.. (2016). Functional divergence of poplar histidine-aspartate kinase HK1 paralogs in response to osmotic stress. Int. J. Mol. Sci. 17 (12), 2061. doi: 10.3390/ijms17122061 27941652 PMC5187861

[B73] Hernàndez-Sebastiá C. Varin L. Marsolais F. (2008). “Sulfotransferases from plants, algae and phototrophic bacteria,” in Sulfur metabolism in phototrophic organisms (Dordrecht: Springer), 111–130.

[B74] Hirschmann F. Krause F. Papenbrock J. (2014). The multi-protein family of sulfotransferases in plants: composition, occurrence, substrate specificity, and functions. Front. Plant science. 5, 556. doi: 10.3389/fpls.2014.00556 PMC4199319 25360143

[B82] Hong Y. Pan X. Welti R. Wang X. (2008). Phospholipase Dα3 is involved in the hyperosmotic response in arabidopsis. Plant Cell. 20 (3), 803–816. doi: 10.1105/tpc.107.056390 18364466 PMC2329935

[B61] Hui-Hui Z. Guang-Liang S. Jie-Yu S. Xin L. Ma-Bo L. Liang M.. (2019). Photochemistry and proteomics of mulberry (Morus alba l.) seedlings under NaCl and NaHCO3 stress. Ecotoxicol. Environ. Saf. 184, 109624. doi: 10.1016/j.ecoenv.2019.109624 31487570

[B17] Hurkman W. J. Tanaka C. K. (1986). Solubilization of plant membrane proteins for analysis by two-dimensional gel electrophoresis. Plant Physiol. 81 (3), 802–806. doi: 10.1104/pp.81.3.802 16664906 PMC1075430

[B40] Jamshidi Goharrizi K. Amirmahani F. Salehi F. (2020). Assessment of changes in physiological and biochemical traits in four pistachio rootstocks under drought, salinity and drought+ salinity stresses. Physiologia plantarum. 168 (4), 973–989. doi: 10.1111/ppl.13042 31670837

[B51] Jamshidi Goharrizi K. Baghizadeh A. Kalantar M. Fatehi F. (2020). Assessment of changes in some biochemical traits and proteomic profile of UCB-1 pistachio rootstock leaf under salinity stress. J. Plant Growth regulation. 39 (2), 608–630. doi: 10.1007/s00344-019-10004-3

[B67] Jander G. Joshi V. (2009). “Aspartate-derived amino acid biosynthesis in arabidopsis thaliana,” in The arabidopsis book/American society of plant biologists, Rockville, MD. vol. 7. 10.1199/tab.0121 PMC3243338 22303247

[B87] Ji T. Li S. Li L. Huang M. Wang X. Wei M.. (2018). Cucumber phospholipase d alpha gene overexpression in tobacco enhanced drought stress tolerance by regulating stomatal closure and lipid peroxidation. BMC Plant Biol. 18 (1), 1–14. doi: 10.1186/s12870-018-1592-y 30547756 PMC6293578

[B79] Jin L. Ouyang N. Huang Y. Liu C. Ruan Y. (2019). Genome-wide analysis of sulfotransferase genes and their responses to abiotic stresses in Chinese cabbage (Brassica rapa l.). PloS One 14 (8), e0221422. doi: 10.1371/journal.pone.0221422 31425555 PMC6699706

[B29] Jungklang J. Saengnil K. Uthaibutra J. (2017). Effects of water-deficit stress and paclobutrazol on growth, relative water content, electrolyte leakage, proline content and some antioxidant changes in curcuma alismatifolia gagnep. cv. Chiang mai pink. Saudi J. Biol. Sci. 24 (7), 1505–1512. doi: 10.1016/j.sjbs.2015.09.017 30294219 PMC6169545

[B93] Kai W. Wang J. Liang B. Fu Y. Zheng Y. Zhang W.. (2019). PYL9 is involved in the regulation of ABA signaling during tomato fruit ripening. J. Exp. botany. 70 (21), 6305–6319. doi: 10.1093/jxb/erz396 31504753 PMC6859720

[B136] Kamireddy K. Sonbarse P. P. Mishra S. Agrawal L. Chauhan P. S. Lata C.. (2021). Proteomic approach to identify the differentially abundant proteins during flavour development in tuberous roots of decalepis hamiltonii Wight & arn. 3 Biotech. 11 (4), 1–19. doi: 10.1007/s13205-021-02714-x PMC7973354 33927964

[B64] Kappachery S. Sasi S. Alyammahi O. Alyassi A. Venkatesh J. Gururani M. A. (2021). Overexpression of cytoplasmic solanum tuberosum glyceraldehyde 3-phosphate dehydrogenase (GAPDH) gene improves PSII efficiency and alleviates salinity stress in arabidopsis. J. Plant Interactions. 16 (1), 398–410. doi: 10.1080/17429145.2021.1962420

[B12] Khoyerdi F. F. Shamshiri M. H. Estaji A. (2016). Changes in some physiological and osmotic parameters of several pistachio genotypes under drought stress. Scientia horticulturae. 198, 44–51. doi: 10.1016/j.scienta.2015.11.028

[B33] Kim J. Liu Y. Zhang X. Zhao B. Childs K. L. (2016). Analysis of salt-induced physiological and proline changes in 46 switchgrass (Panicum virgatum) lines indicates multiple response modes. Plant Physiol. Biochem. 105, 203–212. doi: 10.1016/j.plaphy.2016.04.020 27111258

[B31] Koenigshofer H. Loeppert H.-G. (2019). The up-regulation of proline synthesis in the meristematic tissues of wheat seedlings upon short-term exposure to osmotic stress. J. Plant Physiol. 237, 21–29. doi: 10.1016/j.jplph.2019.03.010 30999074

[B60] Krech K. Ruf S. Masduki F. F. Thiele W. Bednarczyk D. Albus C. A.. (2012). The plastid genome-encoded Ycf4 protein functions as a nonessential assembly factor for photosystem I in higher plants. Plant Physiol. 159 (2), 579–591. doi: 10.1104/pp.112.196642 22517411 PMC3375926

[B45] Lan C.-Y. Lin K.-H. Chen C.-L. Huang W.-D. Chen C.-C. (2020). Comparisons of chlorophyll fluorescence and physiological characteristics of wheat seedlings influenced by iso-osmotic stresses from polyethylene glycol and sodium chloride. Agronomy 10 (3), 325. doi: 10.3390/agronomy10030325

[B78] Leitão I. Leclercq C. C. Ribeiro D. M. Renaut J. Almeida A. M. Martins L. L.. (2021). Stress response of lettuce (Lactuca sativa) to environmental contamination with selected pharmaceuticals: A proteomic study. J. Proteomics. 245, 104291. doi: 10.1016/j.jprot.2021.104291 34089899

[B16] Lichtenthaler H. K. Buschmann C. (2001). Chlorophylls and carotenoids: Measurement and characterization by UV-VIS spectroscopy. Curr. Protoc. Food analytical Chem. 1 (1), F4. 3.1–F4. 3.8. doi: 10.1002/0471142913.faf0403s01

[B110] Li H. Li Y. Ke Q. Kwak S.-S. Zhang S. Deng X. (2020). Physiological and differential proteomic analyses of imitation drought stress response in sorghum bicolor root at the seedling stage. Int. J. Mol. Sci. 21 (23), 9174. doi: 10.3390/ijms21239174 33271965 PMC7729455

[B90] Liu Q. Liu R. Zhou Y. Wang W. Wu G. Yang N. (2022). Phospholipase dδ and H2S increase the production of NADPH oxidase-dependent H2O2 to respond to osmotic stress-induced stomatal closure in arabidopsis thaliana. J. Plant Physiol. 270, 153617. doi: 10.1016/j.jplph.2022.153617 35042010

[B129] Liu Y. Wang X. Dong D. Guo L. Dong X. Leng J.. (2021). Research advances in heterotrimeric G-protein α subunits and uncanonical G-protein coupled receptors in plants. Int. J. Mol. Sci. 22 (16), 8678. doi: 10.3390/ijms22168678 34445383 PMC8395518

[B97] Liu C. Xu Y. Feng Y. Long D. Cao B. Xiang Z.. (2018). Ectopic expression of mulberry G-proteins alters drought and salt stress tolerance in tobacco. Int. J. Mol. Sci. 20 (1), 89. doi: 10.3390/ijms20010089 30587818 PMC6337368

[B89] Liu Q. Zhou Y. Li H. Liu R. Wang W. Wu W.. (2021). Osmotic stress-triggered stomatal closure requires phospholipase dδ and hydrogen sulfide in arabidopsis thaliana. Biochem. Biophys. Res. Commun. 534, 914–920. doi: 10.1016/j.bbrc.2020.10.074 33187643

[B134] Ma Y. Dai X. Xu Y. Luo W. Zheng X. Zeng D.. (2015). COLD1 confers chilling tolerance in rice. Cell 160 (6), 1209–1221. doi: 10.1016/j.cell.2015.01.046 25728666

[B81] Mao G. Wang R. Guan Y. Liu Y. Zhang S. (2011). Sulfurtransferases 1 and 2 play essential roles in embryo and seed development in arabidopsis thaliana. J. Biol. Chem. 286 (9), 7548–7557. doi: 10.1074/jbc.M110.182865 21189252 PMC3045009

[B105] Mao X. Zhang H. Tian S. Chang X. Jing R. (2010). TaSnRK2. 4, an SNF1-type serine/threonine protein kinase of wheat (Triticum aestivum l.), confers enhanced multistress tolerance in arabidopsis. J. Exp. botany. 61 (3), 683–696. doi: 10.1093/jxb/erp331 20022921 PMC2814103

[B111] Marondedze C. Thomas L. Lilley K. S. Gehring C. (2020). Drought stress causes specific changes to the spliceosome and stress granule components. Front. Mol. biosciences. 6, 163. doi: 10.3389/fmolb.2019.00163 PMC6985371 32039234

[B32] Mattioli R. Palombi N. Funck D. Trovato M. (2020). Proline accumulation in pollen grains as potential target for improved yield stability under salt stress. Front. Plant science. 11, 582877. doi: 10.3389/fpls.2020.582877 PMC7655902 33193531

[B54] Mayfield S. P. (1991). Over-expression of the oxygen-evolving enhancer 1 protein and its consequences on photosystem II accumulation. Planta 185 (1), 105–110. doi: 10.1007/BF00194521 24186286

[B36] Mechri B. Tekaya M. Hammami M. Chehab H. (2020). Effects of drought stress on phenolic accumulation in greenhouse-grown olive trees (Olea europaea). Biochem. Systematics Ecology. 92, 104112. doi: 10.1016/j.bse.2020.104112

[B124] Meena M. Divyanshu K. Kumar S. Swapnil P. Zehra A. Shukla V.. (2019). Regulation of l-proline biosynthesis, signal transduction, transport, accumulation and its vital role in plants during variable environmental conditions. Heliyon 5 (12), e02952. doi: 10.1016/j.heliyon.2019.e02952 31872123 PMC6909094

[B96] Miao C. Xiao L. Hua K. Zou C. Zhao Y. Bressan R. A.. (2018). Mutations in a subfamily of abscisic acid receptor genes promote rice growth and productivity. Proc. Natl. Acad. Sci. 115 (23), 6058–6063. doi: 10.1073/pnas.1804774115 29784797 PMC6003368

[B130] Misra S. Wu Y. Venkataraman G. Sopory S. K. Tuteja N. (2007). Heterotrimeric G-protein complex and G-protein-coupled receptor from a legume (Pisum sativum): role in salinity and heat stress and cross-talk with phospholipase c. Plant J. 51 (4), 656–669. doi: 10.1111/j.1365-313X.2007.03169.x 17587233

[B24] Moazzam Jazi M. Ghadirzadeh Khorzoghi E. Botanga C. Seyedi S. M. (2016). Identification of reference genes for quantitative gene expression studies in a non-model tree pistachio (Pistacia vera l.). PloS One 11 (6), e0157467. doi: 10.1371/journal.pone.0157467 27308855 PMC4911069

[B42] Morales M. Munné-Bosch S. (2019). Malondialdehyde: facts and artifacts. Plant Physiol. 180 (3), 1246–1250. doi: 10.1104/pp.19.00405 31253746 PMC6752910

[B75] Most P. Papenbrock J. (2015). Possible roles of plant sulfurtransferases in detoxification of cyanide, reactive oxygen species, selected heavy metals and arsenate. Molecules 20 (1), 1410–1423. doi: 10.3390/molecules20011410 25594348 PMC6272796

[B66] Muñoz-Bertomeu J. Cascales-Miñana B. Alaiz M. Segura J. A. Ros R. (2010). A critical role of plastidial glycolytic glyceraldehyde-3-phosphate dehydrogenase in the control of plant metabolism and development. Plant Signaling Behavior. 5 (1), 67–69. doi: 10.4161/psb.5.1.10200 20592814 PMC2835963

[B37] Naikoo M. I. Dar M. I. Raghib F. Jaleel H. Ahmad B. Raina A.. (2019). “Role and regulation of plants phenolics in abiotic stress tolerance: An overview. Plant signaling molecules, (Cambridge: Woodhead Publishing) 157–168. doi: 10.1016/B978-0-12-816451-8.00009-5

[B80] Nakamura T. Yamaguchi Y. Sano H. (2000). Plant mercaptopyruvate sulfurtransferases: molecular cloning, subcellular localization and enzymatic activities. Eur. J. Biochem. 267 (17), 5621–5630. doi: 10.1046/j.1432-1327.2000.01633.x 10951223

[B59] Nellaepalli S. Ozawa S.-I. Kuroda H. Takahashi Y. (2018). The photosystem I assembly apparatus consisting of Ycf3–Y3IP1 and Ycf4 modules. Nat. Commun. 9 (1), 1–10. doi: 10.1038/s41467-018-04823-3 29934511 PMC6015050

[B4] Ngara R. Ramulifho E. Movahedi M. Shargie N. G. Brown A. P. Chivasa S. (2018). Identifying differentially expressed proteins in sorghum cell cultures exposed to osmotic stress. Sci. Rep. 8 (1), 1–12. doi: 10.1038/s41598-018-27003-1 29875393 PMC5989219

[B119] Ni C. Z. Wang H. Q. Xu T. Qu Z. Liu G. Q. (2005). AtKP1, a kinesin-like protein, mainly localizes to mitochondria in arabidopsis thaliana. Cell Res. 15 (9), 725–733. doi: 10.1038/sj.cr.7290342 16212879

[B117] Nonomura K.-I. Morohoshi A. Nakano M. Eiguchi M. Miyao A. Hirochika H.. (2007). A germ cell–specific gene of the ARGONAUTE family is essential for the progression of premeiotic mitosis and meiosis during sporogenesis in rice. Plant Cell. 19 (8), 2583–2594. doi: 10.1105/tpc.107.053199 17675402 PMC2002623

[B22] Pakzad R. Fatehi F. Kalantar M. Maleki M. (2019). Evaluating the antioxidant enzymes activities, lipid peroxidation and proteomic profile changing in UCB-1 pistachio rootstock leaf under drought stress. Scientia Horticulturae. 256, 108617. doi: 10.1016/j.scienta.2019.108617

[B120] Palmer A. J. Baker A. Muench S. P. (2016). The varied functions of aluminium-activated malate transporters–much more than aluminium resistance. Biochem. Soc. Trans. 44 (3), 856–862. doi: 10.1042/BST20160027 27284052 PMC5264499

[B35] Pál M. Tajti J. Szalai G. Peeva V. Végh B. Janda T. (2018). Interaction of polyamines, abscisic acid and proline under osmotic stress in the leaves of wheat plants. Sci. Rep. 8 (1), 1–12. doi: 10.1038/s41598-018-31297-6 30150658 PMC6110863

[B99] Pandey S. Assmann S. M. (2004). The arabidopsis putative G protein–coupled receptor GCR1 interacts with the G protein α subunit GPA1 and regulates abscisic acid signaling. Plant Cell. 16 (6), 1616–1632. doi: 10.1105/tpc.020321 15155892 PMC490050

[B76] Papenbrock J. Schmidt A. (2000). Characterization of a sulfurtransferase from arabidopsis thaliana. Eur. J. Biochem. 267 (1), 145–154. doi: 10.1046/j.1432-1327.2000.00980.x 10601861

[B100] Park C.-J. Seo Y.-S. (2015). Heat shock proteins: a review of the molecular chaperones for plant immunity. Plant Pathol. J. 31 (4), 323. doi: 10.5423/PPJ.RW.08.2015.0150 26676169 PMC4677741

[B6] Pasaribu S. Basyuni M. Purba E. Hasanah Y. (2021). “The estimated of 18.1 kDa class IV small heat shock protein (sHsp) from hevea brasiliensis using of PHYRE2 and SWISS-MODEL software,” in IOP conference series: Earth and environmental science (Indonesia: IOP Publishing).

[B128] Patel J. S. Selvaraj V. Gunupuru L. R. Kharwar R. N. Sarma B. K. (2020). Plant G-protein signaling cascade and host defense. 3 Biotech. 10 (5), 1–8. doi: 10.1007/s13205-020-02201-9 32355593 PMC7188744

[B25] Pfaffl M. W. Horgan G. W. Dempfle L. (2002). Relative expression software tool (REST©) for group-wise comparison and statistical analysis of relative expression results in real-time PCR. Nucleic Acids Res. 30 (9), e36–e3e. doi: 10.1093/nar/30.9.e36 11972351 PMC113859

[B38] Piwowarczyk B. Tokarz K. Makowski W. Łukasiewicz A. (2017). Different acclimatization mechanisms of two grass pea cultivars to osmotic stress in *in vitro* culture. Acta Physiologiae Plantarum. 39 (4), 1–5. doi: 10.1007/s11738-017-2389-6

[B10] Rahimi B. Afzali M. Farhadi F. Alamolhoda A. A. (2021). Reverse osmosis desalination for irrigation in a pistachio orchard. Desalination 516, 115236. doi: 10.1016/j.desal.2021.115236

[B101] Rahman M. A. Ren L. Wu W. Yan Y. (2015). Proteomic analysis of PEG-induced drought stress responsive protein in TERF1 overexpressed sugarcane (Saccharum officinarum) leaves. Plant Mol. Biol. Reporter. 33 (3), 716–730. doi: 10.1007/s11105-014-0784-3

[B46] Rahneshan Z. Nasibi F. Moghadam A. A. (2018). Effects of salinity stress on some growth, physiological, biochemical parameters and nutrients in two pistachio (Pistacia vera l.) rootstocks. J. Plant Interact. 13 (1), 73–82. doi: 10.1080/17429145.2018.1424355

[B121] Ramesh S. A. Kamran M. Sullivan W. Chirkova L. Okamoto M. Degryse F.. (2018). Aluminum-activated malate transporters can facilitate GABA transport. Plant Cell. 30 (5), 1147–1164. doi: 10.1105/tpc.17.00864 29618628 PMC6002190

[B106] Rampino P. De Pascali M. De Caroli M. Luvisi A. De Bellis L. Piro G.. (2017). Td4IN2: A drought-responsive durum wheat (Triticum durum desf.) gene coding for a resistance like protein with serine/threonine protein kinase, nucleotide binding site and leucine rich domains. Plant Physiol. Biochem. 120, 223–231. doi: 10.1016/j.plaphy.2017.10.010 29065389

[B84] Rodas-Junco B. A. Racagni-Di-Palma G. E. Canul-Chan M. Usorach J. Hernández-Sotomayor S. T. (2021). Link between lipid second messengers and osmotic stress in plants. Int. J. Mol. Sci. 22 (5), 2658. doi: 10.3390/ijms22052658 33800808 PMC7961891

[B23] Sadeghi B. Mirzaei S. Fatehi F. (2022). The proteomic analysis of the resistance responses in tomato during interaction with alternaria alternate. Scientia Horticulturae. 304, 111295. doi: 10.1016/j.scienta.2022.111295

[B83] Saucedo-García M. Gavilanes-Ruíz M. Arce-Cervantes O. (2015). Long-chain bases, phosphatidic acid, MAPKs, and reactive oxygen species as nodal signal transducers in stress responses in arabidopsis. Front. Plant science. 6, 55. doi: 10.3389/fpls.2015.00055 PMC4327526 25763001

[B126] Shan Z. Luo X. Wei M. Huang T. Khan A. Zhu Y. (2018). Physiological and proteomic analysis on long-term drought resistance of cassava (Manihot esculenta crantz). Sci. Rep. 8 (1), 1–12. doi: 10.1038/s41598-018-35711-x 30568257 PMC6299285

[B49] Shayan S. Norouzi M. Vahed M. M. Mohammadi S. A. Toorchi M. (2020). Leaf proteome pattern of two bread wheat varieties under water deficit stress conditions. Curr. Plant Biol. 23, 100146. doi: 10.1016/j.cpb.2020.100146

[B30] Skirycz A. De Bodt S. Obata T. De Clercq I. Claeys H. De Rycke R.. (2010). Developmental stage specificity and the role of mitochondrial metabolism in the response of arabidopsis leaves to prolonged mild osmotic stress. Plant Physiol. 152 (1), 226–244. doi: 10.1104/pp.109.148965 19906889 PMC2799359

[B108] Song Z. T. Liu J. X. Han J. J. (2021). Chromatin remodeling factors regulate environmental stress responses in plants. J. Integr. Plant Biol. 63 (3), 438–450. doi: 10.1111/jipb.13064 33421288

[B104] Sun X.-L. Yu Q.-Y. Tang L.-L. Ji W. Bai X. Cai H.. (2013). GsSRK, a G-type lectin s-receptor-like serine/threonine protein kinase, is a positive regulator of plant tolerance to salt stress. J. Plant Physiol. 170 (5), 505–515. doi: 10.1016/j.jplph.2012.11.017 23276523

[B116] Takashi Y. Kobayashi Y. Tanaka K. Tamura K. (2009). Arabidopsis replication protein a 70a is required for DNA damage response and telomere length homeostasis. Plant Cell Physiol. 50 (11), 1965–1976. doi: 10.1093/pcp/pcp140 19812063

[B3] Toorchi M. Yukawa K. Nouri M.-Z. Komatsu S. (2009). Proteomics approach for identifying osmotic-stress-related proteins in soybean roots. Peptides 30 (12), 2108–2117. doi: 10.1016/j.peptides.2009.09.006 19747515

[B102] Turek I. Wheeler J. Bartels S. Szczurek J. Wang Y. H. Taylor P.. (2020). A natriuretic peptide from arabidopsis thaliana (AtPNP-a) can modulate catalase 2 activity. Sci. Rep. 10 (1), 1–14. doi: 10.1038/s41598-020-76676-0 33184368 PMC7665192

[B91] Urban M. O. Planchon S. Hoštičková I. Vanková R. Dobrev P. Renaut J.. (2021). The resistance of oilseed rape microspore-derived embryos to osmotic stress is associated with the accumulation of energy metabolism proteins, redox homeostasis, higher abscisic acid, and cytokinin contents. Front. Plant Sci. 1161. doi: 10.3389/fpls.2021.628167 PMC8231708 34177973

[B118] Velasquez S. M. Ricardi M. M. Dorosz J. G. Fernandez P. V. Nadra A. D. Pol-Fachin L.. (2011). O-Glycosylated cell wall proteins are essential in root hair growth. Science 332 (6036), 1401–1403. doi: 10.1126/science.1206657 21680836

[B15] Velikova V. Yordanov I. Edreva A. (2000). Oxidative stress and some antioxidant systems in acid rain-treated bean plants: protective role of exogenous polyamines. Plant science. 151 (1), 59–66. doi: 10.1016/S0168-9452(99)00197-1

[B139] Venkatesh J. Kang M.-Y. Liu L. Kwon J.-K. Kang B.-C. (2020). F-box family genes, LTSF1 and LTSF2, regulate low-temperature stress tolerance in pepper (capsicum chinense). Plants 9 (9), 1186. doi: 10.3390/plants9091186 32933000 PMC7570372

[B125] Wang C.-F. Han G.-L. Yang Z.-R. Li Y.-X. Wang B.-S. (2022). Plant salinity sensors: Current understanding and future directions. Front. Plant Sci. 13. doi: 10.3389/fpls.2022.859224 PMC9022007 35463402

[B5] Wang Y. Sang Z. Xu S. Xu Q. Zeng X. Jabu D.. (2020). Comparative proteomics analysis of Tibetan hull-less barley under osmotic stress *via* data-independent acquisition mass spectrometry. GigaScience 9 (3), giaa019. doi: 10.1093/gigascience/giaa019 32126136 PMC7053489

[B138] Wang J. Yao W. Wang L. Ma F. Tong W. Wang C.. (2017). Overexpression of VpEIFP1, a novel f-box/Kelch-repeat protein from wild Chinese vitis pseudoreticulata, confers higher tolerance to powdery mildew by inducing thioredoxin z proteolysis. Plant Science. 263, 142–155. doi: 10.1016/j.plantsci.2017.07.004 28818370

[B85] Wei J. Shao W. Liu X. He L. Zhao C. Yu G.. (2022). Genome-wide identification and expression analysis of phospholipase d gene in leaves of sorghum in response to abiotic stresses. Physiol. Mol. Biol. Plants. 28 (6), 1261–1276. doi: 10.1007/s12298-022-01200-9 35910446 PMC9334518

[B109] Wong M. M. Chong G. L. Verslues P. E. (2017). “Epigenetics and RNA processing: connections to drought, salt, and ABA?,” in Plant stress tolerance (New York: Springer), 3–21. 10.1007/978-1-4939-7136-7_1 28735388

[B92] Xing L. Zhao Y. Gao J. Xiang C. Zhu J.-K. (2016). The ABA receptor PYL9 together with PYL8 plays an important role in regulating lateral root growth. Sci. Rep. 6 (1), 1–13. doi: 10.1038/srep27177 27256015 PMC4891660

[B1] Xiong L. Zhu J. K. (2002). Molecular and genetic aspects of plant responses to osmotic stress. Plant Cell Environment. 25 (2), 131–139. doi: 10.1046/j.1365-3040.2002.00782.x 11841658

[B77] Yamasaki H. Cohen M. F. (2016). Biological consilience of hydrogen sulfide and nitric oxide in plants: gases of primordial earth linking plant, microbial and animal physiologies. Nitric. Oxide 55, 91–100. doi: 10.1016/j.niox.2016.04.002 27083071

[B123] Zandalinas S. I. Fritschi F. B. Mittler R. (2020). Signal transduction networks during stress combination. J. Exp. Botany. 71 (5), 1734–1741. doi: 10.1093/jxb/erz486 31665392

[B2] Zang X. Komatsu S. (2007). A proteomics approach for identifying osmotic-stress-related proteins in rice. Phytochemistry 68 (4), 426–437. doi: 10.1016/j.phytochem.2006.11.005 17169384

[B34] Zegaoui Z. Planchais S. Cabassa C. Djebbar R. Belbachir O. A. Carol P. (2017). Variation in relative water content, proline accumulation and stress gene expression in two cowpea landraces under drought. J. Plant Physiol. 218, 26–34. doi: 10.1016/j.jplph.2017.07.009 28763706

[B135] Zhang X. Gou M. Liu C.-J. (2013). Arabidopsis kelch repeat f-box proteins regulate phenylpropanoid biosynthesis *via* controlling the turnover of phenylalanine ammonia-lyase. Plant Cell. 25 (12), 4994–5010. doi: 10.1105/tpc.113.119644 24363316 PMC3904001

[B7] Zhang C. Shi S. (2018). Physiological and proteomic responses of contrasting alfalfa (Medicago sativa l.) varieties to PEG-induced osmotic stress. Front. Plant Sci. 9, 242. doi: 10.3389/fpls.2018.00242 29541085 PMC5835757

[B127] Zhang Y. Wei M. Liu A. Zhou R. Li D. Dossa K.. (2019). Comparative proteomic analysis of two sesame genotypes with contrasting salinity tolerance in response to salt stress. J. proteomics. 201, 73–83. doi: 10.1016/j.jprot.2019.04.017 31009803

[B50] Zhang F. Zhu G. Du L. Shang X. Cheng C. Yang B.. (2016). Genetic regulation of salt stress tolerance revealed by RNA-seq in cotton diploid wild species, gossypium davidsonii. Sci. Rep. 6 (1), 1–15. doi: 10.1038/srep20582 26838812 PMC4738326

[B52] Zhou G. Yang L.-T. Li Y.-R. Zou C.-L. Huang L.-P. Qiu L.-H.. (2012). Proteomic analysis of osmotic stress-responsive proteins in sugarcane leaves. Plant Mol. Biol. reporter. 30 (2), 349–359. doi: 10.1007/s11105-011-0343-0

[B48] Zhu D. Luo F. Zou R. Liu J. Yan Y. (2021). Integrated physiological and chloroplast proteome analysis of wheat seedling leaves under salt and osmotic stresses. J. Proteomics. 234, 104097. doi: 10.1016/j.jprot.2020.104097 33401000

