# Supplementary material for: Proteomics approach to investigating osmotic stress effects on pistachio
Source: Front Plant Sci. 2023 Jan 25;13:1041649. doi: 10.3389/fpls.2022.1041649 (PMC9907329; doi:10.3389/fpls.2022.1041649)
Supplement: Supplementary file 1 [file Table_1.pdf]

Spot ID: 12

http://www.matrixscience.com/cgi/master_results.pl?file=..%2Fdata%2F20201225%2FFTtASiEES.dat;sessionID=guest_guestsession

Spot ID: 16
http://www.matrixscience.com/cgi/master_results.pl?file=..%2Fdata%2F20201225%2FFTtASiSEh.dat;sessionID=guest_guestsession

Spot ID: 22
http://www.matrixscience.com/cgi/master_results.pl?file=..%2Fdata%2F20201225%2FFTtASiSTS.dat;sessionID=guest_guestsession

Spot ID: 31
http://www.matrixscience.com/cgi/master_results.pl?file=..%2Fdata%2F20201225%2FFTtASiYTe.dat;sessionID=guest_guestsession

Spot ID: 34
http://www.matrixscience.com/cgi/master_results.pl?file=..%2Fdata%2F20201225%2FFTtASiSeE.dat;sessionID=guest_guestsession

Spot ID: 57
http://www.matrixscience.com/cgi/master_results.pl?file=..%2Fdata%2F20201225%2FFTtASiSwS.dat;sessionID=guest_guestsession

Spot ID: 60

http://www.matrixscience.com/cgi/master_results.pl?file=..%2Fdata%2F20201225%2FFTtASiEem.dat;sessionID=guest_guestsession

Spot ID: 64
http://www.matrixscience.com/cgi/master_results.pl?file=..%2Fdata%2F20201225%2FFTtASiYTL.dat;sessionID=guest_guestsession

Spot ID: 88
http://www.matrixscience.com/cgi/master_results.pl?file=..%2Fdata%2F20201225%2FFTtASiYnO.dat;sessionID=guest_guestsession

Spot ID: 102
http://www.matrixscience.com/cgi/master_results.pl?file=..%2Fdata%2F20201225%2FFTtASiSee.dat;sessionID=guest_guestsession

Spot ID: 116
http://www.matrixscience.com/cgi/master_results.pl?file=..%2Fdata%2F20201225%2FFTtASiEmm.dat;sessionID=guest_guestsession

Spot ID: 117

http://www.matrixscience.com/cgi/master_results.pl?file=..%2Fdata%2F20201225%2FFTtASiESR.dat;sessionID=guest_guestsession

Spot ID: 112
http://www.matrixscience.com/cgi/master_results.pl?file=..%2Fdata%2F20201225%2FFTtASiSST.dat;sessionID=guest_guestsession

Spot ID: 113
http://www.matrixscience.com/cgi/master_results.pl?file=..%2Fdata%2F20201225%2FFTtASiSSt.dat;sessionID=guest_guestsession

Spot ID: 114
http://www.matrixscience.com/cgi/master_results.pl?file=..%2Fdata%2F20201225%2FFTtASiSEO.dat;sessionID=guest_guestsession

Spot ID: 119
http://www.matrixscience.com/cgi/master_results.pl?file=..%2Fdata%2F20201225%2FFTtASiSEE.dat;sessionID=guest_guestsession

Spot ID: 122
http://www.matrixscience.com/cgi/master_results.pl?file=..%2Fdata%2F20201225%2FFTtASiSTe.dat;sessionID=guest_guestsession

Spot ID: 126
http://www.matrixscience.com/cgi/master_results.pl?file=..%2Fdata%2F20201225%2FFTtASiSmL.dat;sessionID=guest_guestsession

Spot ID: 133
http://www.matrixscience.com/cgi/master_results.pl?file=..%2Fdata%2F20201225%2FFTtASiSmS.dat;sessionID=guest_guestsession

Spot ID: 137
http://www.matrixscience.com/cgi/master_results.pl?file=..%2Fdata%2F20201225%2FFTtASiYOL.dat;sessionID=guest_guestsession

Spot ID: 147
http://www.matrixscience.com/cgi/master_results.pl?file=..%2Fdata%2F20201225%2FFTtASiEeR.dat;sessionID=guest_guestsession

Spot ID: 162
http://www.matrixscience.com/cgi/master_results.pl?file=..%2Fdata%2F20201225%2FFTtASiYwS.dat;sessionID=guest_guestsession

Spot ID: 165
http://www.matrixscience.com/cgi/master_results.pl?file=..%2Fdata%2F20201225%2FFTtASiSeL.dat;sessionID=guest_guestsession

Spot ID: 173
http://www.matrixscience.com/cgi/master_results.pl?file=..%2Fdata%2F20201225%2FFTtASiEmh.dat;sessionID=guest_guestsession

Spot ID: 174
http://www.matrixscience.com/cgi/master_results.pl?file=..%2Fdata%2F20201225%2FFTtASiSEe.dat;sessionID=guest_guestsession
